# Measuring homoplasy I: comprehensive measures of maximum and minimum cost under parsimony across discrete cost matrix character types

**DOI:** 10.1111/cla.12582

**Published:** 2024-06-25

**Authors:** Jennifer F. Hoyal Cuthill, Graeme T. Lloyd

**Affiliations:** ^1^ School of Life Sciences University of Essex Colchester UK; ^2^ Independent Amble UK

## Abstract

Here, we propose, prove mathematically and discuss maximum and minimum measures of maximum parsimony evolution across 12 discrete phylogenetic character types, classified across 4467 morphological and molecular datasets. Covered character types are: constant, binary symmetric, multistate unordered (non‐additive) symmetric, multistate linear ordered symmetric, multistate non‐linear ordered symmetric, binary irreversible, multistate irreversible, binary Dollo, multistate Dollo, multistate custom symmetric, binary custom asymmetric and multistate custom asymmetric characters. We summarize published solutions and provide and prove a range of new formulae for the algebraic calculation of minimum (*m*), maximum (*g*) and maximum possible (*g*_max_) character cost for applicable character types. Algorithms for exhaustive calculation of *m*, *g* and *g*_max_ applicable to all classified character types (within computational limits on the numbers of taxa and states) are also provided. The general algorithmic solution for minimum steps (*m*) is identical to a minimum spanning tree on the state graph or minimum weight spanning arborescence on the state digraph. Algorithmic solutions for character *g* and *g*_max_ are based on matrix mathematics equivalent to optimization on the star tree, respectively for given state frequencies and all possible state frequencies meeting specified numbers of taxa and states. We show that maximizing possible cost (*g*_max_) with given transition costs can be equivalent to maximizing, across all possible state frequency combinations, the lowest implied cost of state transitions if any one state is ancestral on the star tree, via the solution of systems of linear equations. The methods we present, implemented in the Claddis R package, extend to a comprehensive range, the fundamental character types for which homoplasy may be measured under parsimony using *m*, *g* and *g*_max_, including extra cost (*h*), consistency index (*ci*), retention index (*ri*) or indices based thereon.

## Introduction

Homoplasy includes the phylogenetic manifestation of an evolutionary phenomenon of major interest, the repeated evolution of similar biological traits. This has been a central topic in evolutionary biology and throughout its history has been discussed using a collection of interrelated terms including evolutionary convergence and parallelism (Haas and Simpson, 1946; Hall, 2003). Evolutionary convergence has often been linked to repeated adaptation, while related concepts of evolutionary parallelism have often been linked to genetic, developmental and structural constraints on evolutionary possibilities (Donoghue and Ree, 2000; Conway Morris, 2003; Wake et al., 2011; Powell and Mariscal, 2015; Hoyal Cuthill, 2015a). The measurement of homoplasy therefore underpins efforts to understand the nature and potential outcomes of evolutionary processes.

Quantifying homoplasy is important for several reasons. Homoplasy is known to have severe impacts on the accuracy of phylogenetic reconstruction under parsimony where it can be the cause of statistical inconsistency owing to long branch attraction (Felsenstein, 1978). Homoplasy can also impact model‐based phylogenetic reconstruction using maximum likelihood or Bayesian methods. For example, if the model of character change is violated because it underestimates homoplasy or does not take into account differences in homoplasy levels between data partitions, maximum likelihood and Bayesian methods can also return biased, inaccurate and incorrectly supported phylogenies (Brandley et al., 2009 and references therein). In the extreme, where homoplasy levels are so high that characters are saturated (Wagner, 2000; Hoyal Cuthill, 2015b; Brocklehurst and Benson, 2021), they may not retain sufficient phylogenetic information for phylogenetic reconstruction by any method. It is also notable that, while homoplasy is often viewed primarily as an impediment to phylogenetic reconstruction (as described above), potentially homoplastic characters can still provide valuable information for phylogenetic analysis. In particular, character data with homoplasy can be decisive, providing information for the evaluation of possible phylogenetic trees (Goloboff, 1991), and homoplastic character states may still act as local synapomorphies providing strong support for parts of a tree (Kälersjö et al., 1999).

Measures of homoplasy can, therefore, give indications of tree quality and are widely used as tree or clade support metrics. Homoplasy measures are also used to compare, as sources of phylogenetic information, different data types or partitions such as morphology and molecules, or cranial and postcranial characters (Sánchez‐Villagra and Williams, 1998; Mounce et al., 2016). Some phylogenetic methods also use measures of homoplasy to directly improve phylogenetic reconstruction, for example by the consideration of homoplasy during the tree inference process (Goloboff et al., 2008a and references therein).

Almost all parsimony‐based homoplasy metrics depend (implicitly or explicitly) on first establishing the minimum (*m*) and/or maximum (*g*) possible cost a given character could have under maximum parsimony optimization. These bounds then provide a context for the number of steps (*s*) that character is inferred to have taken on a preferred topology. The value of *s* may be calculated either where the same set of characters are used to infer the phylogeny and homoplasy levels (where *s* is the length of the most parsimonious trees) or where separate character partitions are used, for example if homoplasy is reconstructed among morphological characters on a molecular tree (e.g. Callender‐Crowe and Sansom, 2022). Even where tree reconstruction is performed using other methods, parsimony‐based measures can therefore be useful for the quantification of homoplasy, across a range of character types for example morphology, behaviour, recurrent DNA or RNA mutation (e.g. in SARS‐CoV‐2, van Dorp et al., 2020), gene function (Mendler et al., 2019) or gene regulation.

The simplest measure of homoplasy is the number of extra steps (*h = s* − *m*), over the minimum possible (Camin and Sokal, 1965), something Fisher (1992) termed “parsimony debt” in a stratocladistic context. Perhaps the most commonly used homoplasy measure, the consistency index (Kluge and Farris, 1969), *CI* (character *ci* = *m*/*s*), also depends on first establishing the minimal value, and both the retention index, *RI* (character *ri* = (*g* − *s*)/(*g* − *m*)) and rescaled consistency index, *RCI* (Farris, 1989), require knowing both the minimal *and* maximal values. When maximum and minimum steps are known, a further use is the identification of parsimony uninformative characters, for which *m* = *g* (Farris, 1989).

However, even for parsimony analysis of discrete phylogenetic characters, where the greatest research effort has been applied, we show, in relation to a survey of character types from the literature (Appendix A), that there remain some character types for which minimum (*m*) and maximum (*g*) bounds on parsimony steps (given a number of taxa *t*, number of states *n* and implicit or explicit transition cost matrix), and hence their measurable homoplasy, have not previously been implemented in common software such as PAUP* (Swofford, 2003) and TNT (Goloboff et al., 2008b; Goloboff and Catalano, 2016; Goloboff and Morales, 2023), or their effects explored. Furthermore, to our knowledge, maximum *possible* bounds (*g*_max_) have only previously been determined for the simplest characters (specifically characters in which all transitions between different states have cost 1 (Mickevich, 1978; Steel and Penny, 2006; Hoyal Cuthill et al., 2010)) and we are not aware of any pre‐existing software implementation for calculating *g*_max_. Explicit mathematical proofs for maximum steps, in particular, have previously been explored mainly among the simplest character types, such as unordered characters where all costs of transition between different states are equal to 1 (Hoyal Cuthill et al., 2010).

The maximum possible cost and minimum cost determine the maximum homoplasy that can be shown by common homoplasy measures (Hoyal Cuthill et al., 2010), including extra steps and the consistency index (maximum extra steps *h*_max_ = *g*_max_ − *m*, minimum consistency index *ci*_*min*_ = $m/g_{max}$). Comparisons with *g*_max_ and *g* could also be applied to the retention index of Farris (1989) (*ri* = (*g* − *s*)/(*g* − *m*)) for example via its complement, the distortion coefficient (*d* = (*s* − *m*)/(*g* − *m*)), to give a minimum baseline of *d*_*min*_ = (*s* − *m*)/(*g*_max_ − *m*).

Custom transition cost matrices, in which state transition costs are explicitly defined for a given character, enable the incorporation of models, hypotheses or prior information about the process of evolution in the estimation of tree length and quantification of homoplasy within the context of parsimony. Such concepts have a long history within phylogenetics, including the formalization of early evolutionary hypotheses such as Dollo's law, sometimes defined today as irreversibility of trait loss (Wiens, 2011) but, in cladistic software, often treated as a ban on repeated state gains, i.e. “convergent transitions” (Semple and Steel, 2003, p. 68), introduced below (e.g. Swofford and Olsen, 1990; Swofford, 2003). As new molecular, developmental and structural information emerges, this adds to the wide range of evidence for variable probabilities of different evolutionary transitions, showing for example that evolutionary losses may sometimes be more probable than gains (Wake et al., 2011), as has been argued for complex traits such as phasmid wings (Trueman et al., 2004) and in cases of conserved genetic regulatory mechanisms (for example in *Drosophila* pigmentation; Hughes et al., 2020).

Here we provide methods for the measurement of minimum steps (*m*), maximum steps (*g*) and the maximum possible steps (*g*_max_) any character could show given its specified numbers of taxa and states, and its transition costs. These are provided for a full range of cost matrix character types (listed in full in the “Appendix A”) including character types for which some implemented or described bounds on homoplasy were previously unavailable (with a full list of new and previously available implementations provided in the “Appendix A”).

We provide parsimony measures defined for discrete characters (which have formed the basis for the majority of fundamental theory in the definition and measurement of homoplasy). We note, also, that such concepts and measures of homoplasy among discrete characters are also of potential interest for considerations of continuous characters, to which they have in some cases been extended (for example, see Maddison, 1991; Stayton, 2006; Klingenberg and Gidaszewski, 2010; Arbuckle et al., 2014, Lloyd, 2018, Hoyal Cuthill et al., 2019). In discussion of applications to real data, we primarily consider cost matrices with integer values (or sometimes also infinity), which have also been referred to as step matrices (Swofford and Maddison, 1992). All 4,467 morphological and molecular character matrices surveyed have integer (or infinite) costs, although our algebraic approach (described below), for example, could also be applied to non‐integer costs. Surveyed character matrices are listed at graemetlloyd.com, and represent an expanded version of the database used by Wright et al. (2016) and Wright and Lloyd (2020).

Below, we define parsimony measures of minimal and maximal evolution (*m*, *g* and *g*_max_), and hence homoplasy, across the considered discrete character types and provide details for their implementation, either where documented in PAUP*, TNT, or in novel implementation in the R package Claddis (Lloyd, 2016, 2018; github.com/graemetlloyd/Claddis). We achieve this aim by: (i) constructing a comprehensive classification of discrete character types; (ii) providing empirical examples for each type; (iii) describing how minimal and maximal values may be calculated in theory (including novel solutions) for each type; and (iv) showing how these can be calculated in practice using PAUP*, TNT or Claddis. We also note cases where previously existing software could not calculate these values for every character type. In this paper we make the following assumptions: (i) topologies are considered rooted; (ii) polytomies are considered hard; (iii) reversals and convergent transitions are as defined in Semple and Steel (2003); (iv) every state defined in the cost matrix is actually present in at least one tip; (v) for some characters, gains and losses are defined by a numbered sequence (i.e. 0 to 1 is a gain, 2 to 1 is a loss); (vi) state graph representations are those that minimize total length; (vii) no characters are missing or inapplicable (e.g. see Maddison, 1993); (viii) no characters are uncertain or polymorphic (e.g. see Swofford and Maddison, 1992); and (ix) unless otherwise stated for a given character type (e.g. Dollo and irreversible characters), we assume that there are no pre‐existing constraints on which state may be ancestral, such that all states are available for consideration as a potentially optimal ancestral state (see also discussion below).

### Cost matrices and graph representations

Cost matrices are square matrices with rows (*i*) and columns (*j*) that represent the cost (*c*_*i,j*_) of transition between those states. Formally such costs are often measured in “steps”, but as this refers strictly to integer values we follow Swofford and Maddison (1992) in preferring the general term “cost” as this is inclusive of any off‐diagonal positive value. By convention, rows are considered the “from” state in the transition and columns the “to” state: *c*_*i,j*_ is the cost of going from state *i* to state *j*. Explicit cost matrix representations are rarely used in cladistic datasets. However, all common character types can be represented using a cost matrix, including binary, linear ordered multistate (Wagner, 1961; Farris, 1970) or unordered multistate character types (Fitch, 1971). Indeed, cost matrices are inclusive of all types of cladistic character (Swofford and Maddison, 1992) with limited exceptions, such as Dollo characters, where additional information is typically required (Swofford and Olsen, 1990).

**Fig. 1:**
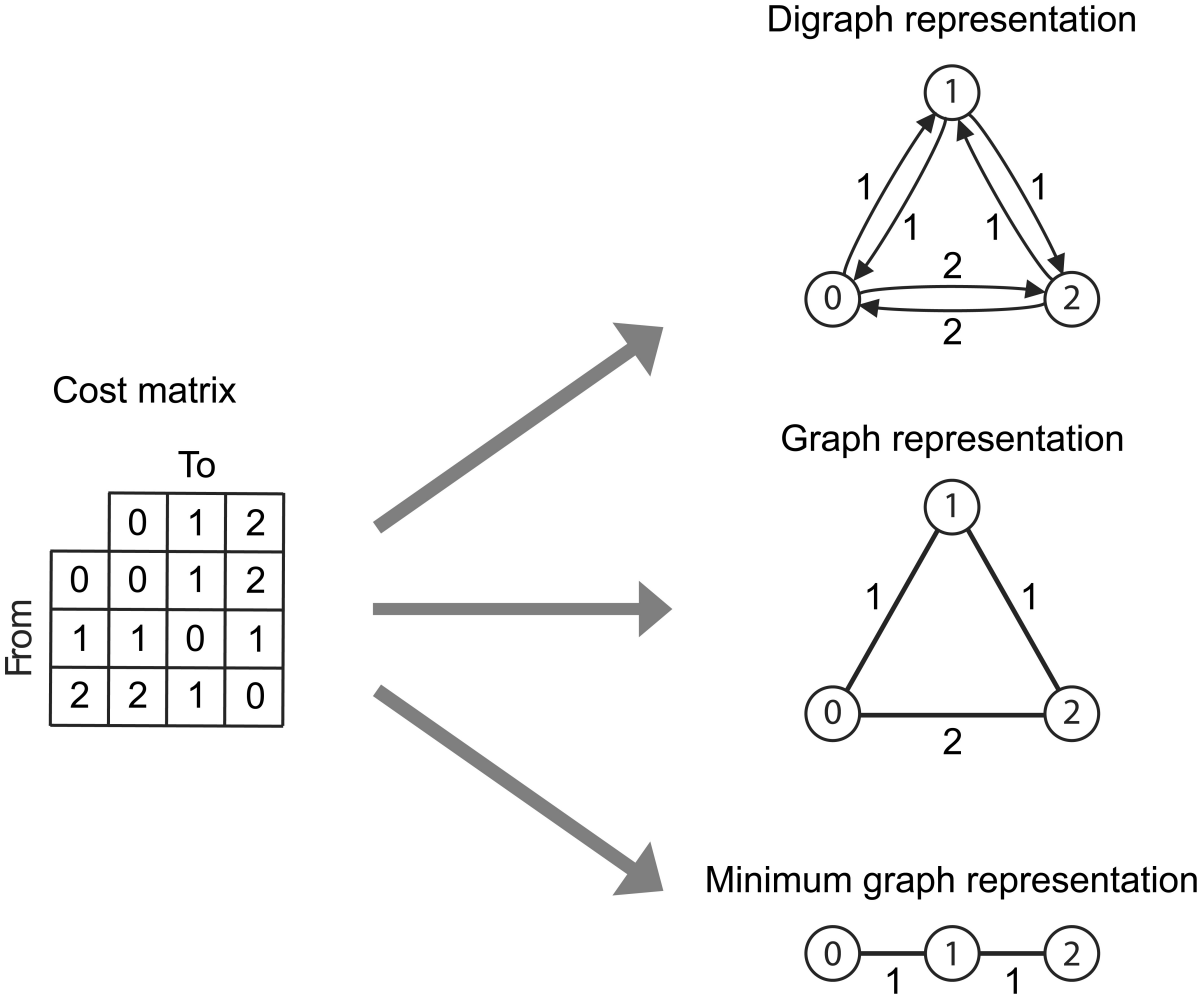
Cost matrices and corresponding graph representations. The cost matrix (at left) represents a three‐state standard linear ordered character—a Type IV character, see Fig. A1 and “Appendix A”. A “complete” digraph (at top right) shows every possible arc (excluding loops) that could be interpreted from the cost matrix. Because, in this case, all costs are symmetric, a digraph representation is not necessary. Middle right shows a “complete” graph representation instead, with a single edge (replacing the two arcs of the digraph) connecting each vertex. However, in practice, the correct graph representation (at bottom right) is the one that also removes any edges with a direct cost that is equal to some indirect transition (i.e. 0–2, here). This means in practice a direct path between states 0 and 2 is not permitted (i.e. the character is considered ordered *sensu* Slowinski, 1993, but not additive *sensu* Goloboff, 2022a).

Any cost matrix can also be represented as a directed graph (a “digraph”), see Fig. 1. However, we must first consider two key differences between a cost matrix and a graph representation. First, for any cost matrix where all transition costs are *symmetric*, the digraph can be replaced with a simpler, undirected graph. This does not change the information content itself, but can be important when considering graph theory solutions or proofs for bounds. Second, a cost matrix may include transitions that would logically be missing or pruned from a digraph. For example, any transition cost of infinity may be described graphically by the lack of an edge, as it would never be traversed by a parsimony algorithm (as any finite cost is lower than infinity). Indeed, the purpose of an infinite cost in a cost matrix is to preclude a specific transition (Swofford and Maddison, 1992). Any direct edge that is of equal cost to an indirect transition, e.g. the 0–2 transition in Fig. 1, is effectively redundant and can also be pruned, producing the *minimal* graph representation (Fig. 1). NB: Direct costs that are larger than an indirect cost are not permitted (by the triangle inequality, see Rule 7, below, and Maddison and Maddison (1992), Maddison (1993) and Wheeler (1993)).

## Rules defining a valid cost matrix for a cladistic character under maximum parsimony

To the best of our knowledge nobody has formally defined what restrictions can be placed on a discrete cost matrix for a cladistic character for it to be considered valid. By valid here we mean only the mathematical limits of validity, not what a systematist may practically consider, which may be more restrictive. Aside from the description above, we identify the following seven rules a cost matrix—and hence a discrete character—must meet to be considered valid:

1. The cost matrix representation must be complete (i.e. a cost must be assigned to every possible transition).
2. The diagonal must always be 0 (i.e. there must be no cost for remaining in the same state)—loops in state graphs are not permitted.
3. The off‐diagonal must always be positive (i.e. there must always be *some* cost for transitioning to a different state).
4. The state graph representation of the cost matrix must be (at least weakly) connected.
5. A maximum of one column can contain all infinite transition costs (excluding the diagonal), i.e. a maximum of one state can be restricted to only appear as a “from” state as by necessity this will be the forced root state and there can only be at most one forced root—the state digraph (such a character must have asymmetric costs and hence a digraph representation) must have single‐source reachability.
6. Similarly, one row must contain no infinite transition costs if all other rows are assigned all infinite costs (excluding the diagonal) (i.e. a maximum of *n* − 1 states can be restricted to only appear as a “to” state as by necessity there must be a root state and this must have access to each of these “to” states).
7. There must be no lower cost indirect state‐to‐state route than the *direct* transition cost (i.e. all transition costs must be self‐consistent) (Maddison and Maddison, 1992; Maddison, 1993; Wheeler, 1993).

**Fig. 2:**
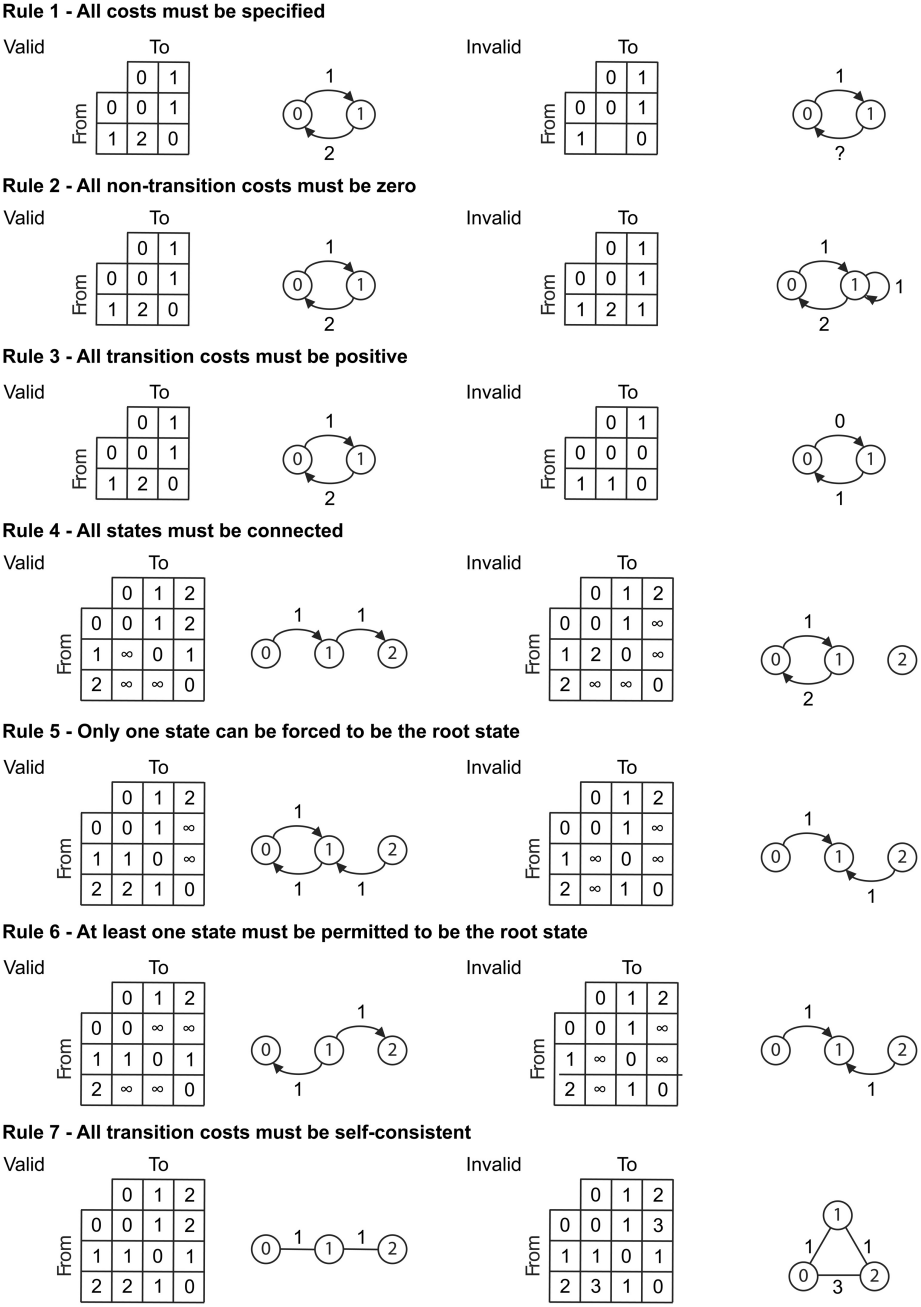
Rules defining a valid cost matrix and associated state (di)graph with examples of valid and invalid cost matrices. For Rule 7 the invalid cost is the direct transition between states 0 and 2 (cost = 3) as a lower cost indirect route is possible via state 1 (cost = 2).

We consider rules 1–3 to be relatively straightforward and in essence mean that the diagonal of a cost matrix has no degrees of freedom, whereas the off‐diagonal has *n* ^2^ − *n* degrees of freedom, albeit with some other limits dictated by the other rules. Rules 4–6 can be generalized such that the (directed) state graph must be both *connected* and *mappable*. By connected we mean that no character state (or subgraph of character states) are isolated from the other state(s). Mappable means mappable to a valid phylogenetic tree (see, e.g. Semple and Steel, 2003 for graph theoretic definitions of phylogenetic trees). For example, a digraph with two “roots” would not be mappable to a phylogenetic tree. Rule 7 was independently suggested by Maddison and Maddison (1992) and Wheeler (1993). Both PAUP* and TNT already automatically correct cost matrices that break Rule 7 by using a triangle inequality check. Examples of valid and invalid cases for Rules 1–7 are shown in Fig. 2.

In practice, we do not expect many empirically applied cost matrices to actually break any of these rules, but consider them here both to explicitly state the problem and facilitate formal mathematical proofs.

## Character types and their minimal and maximal costs under maximum parsimony

### Classification of character types

In this paper we identify 12 different character types, classified in relation to calculating *m*, *g* and *g*_max_, with specific considerations and solutions listed in the “Appendix A”. First, we provide an overview of general solutions and specifics for the most common (or otherwise important) character types.

### Minimum cost

The minimum cost (*m*) which can be achieved for a character with a given number of taxa (*t*), states (*n*) and a specified cost matrix (giving the costs of transition between each pair of states) is the minimum possible cost (also called length or steps) of that character on a most parsimonious tree. Previously Maddison (1989) proposed two algorithms that can be adopted to find a fully resolved tree of length *m* under maximum parsimony for both Fitch (1971; unordered, or Type III characters, see “Appendix A”) and Wagner (1961; linear ordered, or Type IV characters, see “Appendix A”) parsimony. Either algorithm could also be used to find minimum length trees for symmetric binary characters (Type II characters, see “Appendix A”). However, these algorithms are not extensible (generalizable) to the other characters we consider here and instead we propose below a more general approach that can be used to calculate *m* directly.

### General algorithm 1: minimum cost, *m* for cost matrix characters

Any character represented as a cost matrix meeting Rules 1–7 (above) can also be represented as a single minimal (di)graph representation (Fig. 1). For example, the linear ordered character in Fig. 1 has no edge connecting states 0 and 2. This assumption is implicit in cladistic practice but we make it explicit here for the purpose of interpreting cost matrices as state (di)graphs.

For any such state (di)graph there must be a minimum spanning tree (for a graph) or minimum‐weight spanning arborescence (for a digraph) that connects all sampled states and the length of this tree is the minimum cost, *m*. We prove this proposition below (Proof 1).

Proof 1 Any valid state graph must be (weakly) connected (Rule 4) and have at least one vertex that *may* be a root (Rule 6) and no more than one vertex that *must* be a root (Rule 5). In graph theory terms, this means the state (di)graph must have single‐source, multiple‐target reachability—at least one vertex (the root) can reach all other vertices (the tips). As such, the state (di)graph has a minimum spanning tree or minimum‐weight spanning arborescence, and this can be found using established algorithms (Kruskal, 1956; Edmonds, 1967) as specified below.

It only remains to be shown that this connected graph may also be considered as a valid rooted phylogenetic tree. This can be done by following four steps:

For a symmetric character:

1. Find a minimum spanning tree connecting all distinct states present among the tips using Kruskal's algorithm (Kruskal, 1956) and root this tree on any vertex represented among the tips. As costs are symmetric this rooting cannot change the length (total cost) of the tree. The upper bound on the runtime (i.e. algorithmic complexity) of Kruskal's algorithm is known to be, in big‐O notation, *O*(*E* log *n*), where *E* is the number of graph edges and *n* is the number or nodes (vertices) (Kruskal Jr., 2021). In our case, *n* is given by the number of states of a character. For a symmetric character, the appropriate maximum number of edges is that of a complete undirected graph. This is given by *E* = *n*(*n* − 1)/2. Expressed in terms of *n*, the algorithmic complexity of the Kruskal algorithm is then *O*(*n* ^2^ log *n*).

For an asymmetric character:

1. Find a minimum‐weight spanning arborescence connecting all distinct states present among the tips using Edmonds’ algorithm (Edmonds, 1967). An arborescence is rooted by definition and so the root and hence the directionality of change are already determined. The time complexity of Edmonds’ algorithm is *O*(*nE*) (Böther et al., 2023). For a symmetric character, the maximum number of directed edges (arcs) is that of a complete directed graph. This is *E* = *n*(*n* − 1). This gives an algorithmic complexity for the Edmonds algorithm of *O*(*n* ^3^).

Regardless of whether the character is symmetric or asymmetric, after step 1 a rooted digraph is generated and now the same next three steps can be used:

1. For each distinct state present among the tips, create a star graph, *S*_*k*_, where *k* is the frequency (count) of that state among the tips. Root the graph on its single internal node, creating a digraph, and assign the distinct state to both the single root and every tip. These digraphs represent rooted subtrees and under Rule 2 their lengths must be 0.
2. Next attach each subtree generated in step 2 to the minimum spanning digraph created in step 1 by joining each subtree root to its corresponding minimum spanning digraph vertex with a single arc (i.e. state 0 to state 0, state 1 to state 1 etc.). As this edge will have the same state at both ends under Rule 2 this will not add to the length of the resulting digraph.
3. Finally, check for vertices where in‐degree = out‐degree = 1. These are redundant and should be removed to create a valid phylogenetic tree. Such removals cannot affect the length of the tree as either:
  - the removed vertex, its source vertex and its target vertex are all assigned the same state, meaning two arcs of weight 0 are replaced by a single arc of weight 0 for zero net change in length; or
  - the source vertex and target vertex are only connected by an unsampled intermediate vertex (the removed vertex). The sum of these two arcs must be equal to the single replacement arc under Rule 7, resulting in zero net change in length.

The resulting digraph is thus both a valid rooted phylogenetic tree and of minimum length. We can further show that any minimum length rooted phylogenetic tree is minimal, as by collapsing all branches (arcs) of weight 0 what remains will be a minimum‐weight spanning arborescence.

This completes the proof, but if desired, a fully bifurcating minimum length tree can also be generated by adding a fifth step:

1. Check for any polytomous vertices, i.e. those with an out‐degree >2. For each such vertex, prune this vertex from the tree and create a new vertex in its place. Randomly choose one arc emerging from the original vertex and attach this to the new vertex instead. Then add a new arc connecting the new vertex to the original vertex. As the new vertex will always be assigned the same state as the old vertex no arcs from the original tree change weight. Additionally, as the new arc connects two vertices of the same state this will have weight 0 (under Rule 2). Repeat this process until no polytomous vertices remain.

The minimum cost for a general cost matrix character may be calculated this way, including ordered, unordered, irreversible and all other non‐Dollo (Type VIII and IX) characters (“Appendix A”). This algorithm is implemented in Claddis as the find_stategraph_minimum_span function. Furthermore, we provide equivalent formulae for analytic calculation of minimum steps for several specific character types (I–X), including Dollo characters (Type VIII and IX) (Table 1; “Appendix A”). In the “Appendix A” we provide proofs of the applicability of these formulae to these specific character types. It can be seen that the formulae covering these character types are essentially equivalent to the formula *w*(*n* − 1), where *w* is a representative, allowed transition cost from the cost matrix (as specified in Table 1). The characters to which this applies share cost matrix properties of equal direct allowed transition costs and representability by an undirected graph, such that the Kruskal algorithm is applicable for determination of *m* via the length of the minimum spanning tree. Since the minimum spanning tree is of length *n* − 1 (Kruskal, 1956), these properties mean that *m* can be calculated by *w*(*n* − 1).

**Table 1:** Summary of methods of calculation for minimum and maximum parsimony steps among the 12 classified character types

Type	Description	Minimum steps (*m*)		Maximum character steps (*g*)		Maximum possible steps (*g*_max_)	
		General algorithm	Specific formula	General algorithm	Specific formula	General algorithm	Specific formula
I	Constant	1	*m* = 0	2	*g* = 0	3	*g*_max_ = 0
II	Binary symmetric	1	If *c*_0,1_ = *c*_1,0_ = *w*, *m* = *w*(*n* − 1) = *w*(2–1) = *w*	2	If *c*_0,1_ = *c*_1,0_ = *w*, *g* = *w*(*t* − *F*)	3	If *c*_0,1_ = *c*_1,0_ = *w*, *g*_max_ = *w*(*t* − ⌈*t*/2⌉); (eq. (26), above)
III	Multistate unordered symmetric	1	If *c*_0,1_ = *c*_1,0_ = *w* *m* = *w*(*n* − 1)	2	If *c*_0,1_ = *c*_1,0_ = *w*, *g* = *w*(*t* − *F*)	3	If *c*_0,1_ = *c*_1,0_ = *w*, *g*_max_ = w(*t* − ⌈*t*/*n*⌉)
IV	Multistate linear ordered symmetric	1	*m* = *c*_0,1_ + *c*_1,2_ + ⋯ + *c*_*n−*3,*n−*2_ + *c*_*n−*2,*n*−1_ = (*n* − 1)(*c*_0,1_) = *w*(*n* − 1) (eq. (A1)	2	No	3	*g*_max_ = $w(\lfloor \left(n,-,3\right)+\left(t-\left(n-2\right)\right)$ $\times \left(\left(n-1\right)/2,),\rfloor \right)$ (eq. (A2))
V	Multistate non‐linear ordered symmetric	1	*m* = *w*(*n* − 1) (eq. (A1))	2	No	3	No
VI	Binary irreversible	1	$m=c_{0,1}$ = (*n* − 1)(*c*_0,1_) = *w*(*n* − 1) (eq. (A3))	2	$g=yc_{0,1}$ (eq. (A4))	3	$g_{\max}=c_{0,1}\left(t-1\right)$ (eq. (A5))
VII	Multistate irreversible	1	*m* = *c*_0,1_ + *c*_1,2_ + ⋯ + *c*_*n*−3,*n*−2_ + *c*_*n*−2,*n*−1_ = (*n* − 1)(*c*_0,1_) = *w*(*n* − 1) (eq. (A1))	2	*g* = *yc*_0,1_ + *zc*_0,2_ +, …, $f_{n-1}$ *c*_0,*n*−1_ (eq. (A13))	3	$g_{\max}=\left(t-n+1\right)c_{0,n-1}$ $+c_{0,n-2},\ldots ,c_{0,1}$ (eq. (A23))
VIII	Binary Dollo	1	If *c*_1,0_ = 1, *m* = 1		If *c*_1,0_ = 1, *y* = 1, g=1 (eq. (A29)) If *c*_1,0_ = 1, *y* > 1, g=x (eq. (A30)), *D* marginally < *x*		If *t* = *n*, $g_{\max}=c_{1,0}\left(t-1\right)$ If *t* > *n*, $g_{\max}=c_{1,0}\left(t-2\right)$ (eq. (A31)),
IX	Multistate Dollo	1	If *c*_1,0_ = 1, *m* = *n* − 1		*g* = *c*_*a*,0_ *f*_0_ + *c*_*a*,1_ *f*_1_ +, …, *c*_*a*,*n*−1_ *f*_*n*−1_ (eq. (A49)) With *D* marginally < $t-f_{n-1}$ (see eq. (A48))		If *t* < *n* + 1, *g*_max_ = (*n* − 2)(*t* − *n* + 1) + $\frac{1}{2}$(*n* − 2)(*n* − 3) + 1 (eq. (A53)) If *t* > = *n* + 1, *g*_max_ = (*n* − 1)(*t* − *n*) + $\frac{1}{2}$(*n* − 1)(*n* − 2) (eq. (A54))
X	Multistate custom symmetric	1	No	2	No	3	No
XI	Binary custom asymmetric	1	No	2	No	3	(eq. (26))
XII	Multistate custom asymmetric	1	No	2	No	3	No

The general algorithms, outlined above, are: 1, calculation of minimum cost *m* by minimum spanning tree optimization on the cost matrix graph; 2, calculation of maximum character steps *g* by matrix mathematics equivalent to parsimony optimization on the star tree; and 3, guaranteed calculation of maximum possible character steps *g*_max_ (meeting conditions (i)–(iii), of integer state frequencies meeting *n* and *t*) by exhaustive search across possible character state frequencies. *n* denotes the number of represented character states, *t* denotes number of taxa with coded states (not including missing or inapplicable), *w* denotes a specified representative cost, *F* denotes the number of taxa with the most frequent state, *x* denotes the frequency (number of copies among the taxa) of the first state, *y* denotes the frequency of the second state, *z* denotes the frequency of the third state, $f_{n-1}$ is the frequency of the *n* − 1th state, costs are denoted in the form *c*_0,1_ for example indicating the cost of transition from state 0 to state 1, *D* denotes a Dollo penalty and subscript *a* denotes the optimally ancestral state under the Dollo cost penalty. For further details including definitions, proofs and references see the list of individual character type treatments below (Appendix A). R implementations of these formulae are available at github.com/graemetlloyd/Claddis.

### Applicability of star tree optimization as an upper bound on evolution

In the following sections, our aim will be to specify bounds on maximum cost under maximum parsimony. To this end, in common with other references (e.g. Maddison and Maddison, 2003; Swofford, 2003), we make use of optimization on the star tree, or conceptually related algorithms and formulae. The length of the star tree (*g*) is not exceeded by the most parsimonious character cost (*s*) on any possible more resolved phylogenetic tree for the same character (for proofs see discussion in Farris, 1989; Goloboff, 2022b). Our methods for calculation of evolutionary limits are therefore applicable to cases in which star tree optimization is appropriate. A star tree, for a given number of taxa, consists of a single internal, ancestral node, connected by branches (edges) to the external, tip nodes. We note, therefore, that on a star tree all transitions have the *same* starting point so that, if this state is considered ancestral, reversals are not possible on the star tree itself (although it remains possible that there may, in specific cases, be bifurcating trees of the same length where reversal is implied).

### Maximum cost

Maximum cost *g* is the maximum cost for a given character (i.e. for a given character state frequency distribution, meaning the counts of each character state) under maximum parsimony optimization on the star tree. Maximum possible cost *g*_max_ (following notation of Hoyal Cuthill et al., 2010) is the maximum cost a character with *t* taxa and *n* states could possibly exhibit when reconstructed most parsimoniously on the star tree. In other words, *g*_max_ is the maximum value of *g* across all possible distributions of *n* states among *t* taxa (Hoyal Cuthill et al., 2010 and references therein). Here, for comparability with the majority of real cladistic characters, we specify additionally that *g*_max_ should be a cost value that is (i) achievable with integer character state frequencies, and that for a specified number of taxa and character states (ii) the minimum frequency of each state is 1 and (iii) the frequencies of the *n* states sum to the number of taxa *t*, with conditions (i) and (iii) together implying condition (iv) that the maximum frequency of each state is the specified number of taxa minus one fewer than the number of character states. We apply these conditions because, as will be shown below, treating the search for maximum possible steps as an algebraic maximization problem can show that one or more of the state frequencies that correspond to the unconstrained maximum cost is a non‐integer real number, 0 or a negative integer. In contrast, a real cladistic character for *t* taxa and *n* existing states, with each taxon holding one state, would have a maximum parsimony cost restricted to values achievable under conditions (i)–(iii) (although we note this assumes *t* ≥ *n* which might not always be applicable, for example for gap‐weighted characters; Thiele, 1993). We present below, and implemented in the Claddis R package, both an exhaustive search algorithm (general algorithm 3) and rapidly calculable formulae for several specific character types (Table 1; “Appendix A”) to find such values of *g*_max_, which meet conditions (i)–(iv).

#### Maximum possible cost among unordered characters

For unordered characters (but not necessarily general cost matrix characters, including ordered characters, see below), maximum possible cost (*g*_max_) will occur in the maximally balanced distribution of *n* states among *t* taxa (Mickevich, 1978; Steel and Penny, 2006; Hoyal Cuthill et al., 2010). Specifically, for *t* taxa and *n* states (that are actually represented among those taxa), the most balanced distribution of those states possible for an unordered character gives *g*_max_(*t*,*n*) = *t* − ⌈*t*/*n*⌉, where ⌈*t*/*n*⌉ = the smallest integer ≥ *t*/*n*, and is equal to the lowest possible number of taxa with any one most frequent state (*F*_min_) (Hoyal Cuthill et al., 2010). Any given character might have a less balanced distribution than the maximal one (even with the same number of taxa and states), in which case that character would show a capacity for homoplasy lower than the maximal one (such that *g* will be less than *g*_max_). *g* (and *g*_max_) are usually described as maximum steps but, for unordered characters, with transition costs each equal to 1, we can also think of them as the maximum numbers of homoplastic transitions or the maximum numbers of states that could be derived by homoplasy on a tree.

### General algorithm 2: maximum cost (*g*) among general cost matrix characters

#### Algorithmic calculation of maximum cost (*g*)

Values of *g* can be computed by maximum parsimony optimization of character evolution on a star tree (Maddison and Maddison, 2003; Swofford, 2003). We describe here an algorithm which gives an equivalent value of *g* to that on the star tree but is based more directly on the cost matrix.

#### Pseudocode description of the algorithmic calculation of *g*

Take a given cost matrix and make a corresponding row of observed frequencies of the states for a given character. For convenience of further calculation, this row can optionally be vertically duplicated to make a matrix of the same size as the cost matrix.

1. Multiply the frequency of each other state by the cost of transition from the evaluated state to each other state. This takes the product for each row of the cost matrix. Where the frequencies have been duplicated to a matrix of the same size as the cost matrix, these two matrices can simply be multiplied together elementwise.
2. Sum each row in the product matrix, giving the sum cost of evolving each state other than that corresponding to the given row.
3. The minimum row sum is *g*, giving the lowest possible transition cost of evolving all states other than the most parsimoniously reconstructed ancestral state (the one with lowest cost).

**Fig. 3:**
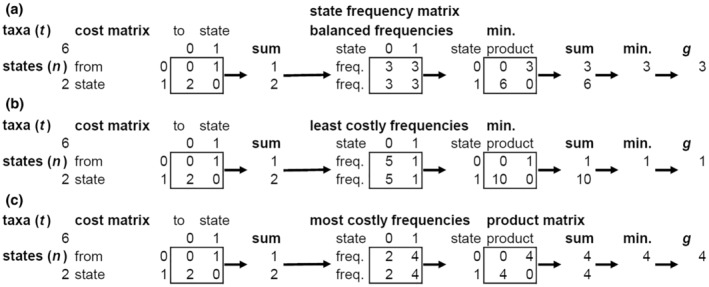
Examples illustrating the algorithmic calculation of maximum homoplasy, *g*. Examples of state frequency distributions for this combination of *t* (number of taxa) and *n* (number of states) (a) balanced, (b) least costly given *t* and *n* and (c) most costly. This example uses a binary asymmetric cost matrix with two states for six taxa.

The above algorithm (worked example, Fig. 3a) is conceptually equivalent to taking each state in turn to evaluate as potentially ancestral on the star tree and then selecting as optimally ancestral the state which gives the lowest total cost of deriving each of the observed character states at their observed frequencies. Since the algorithm operates over *n* vectors, each of which scales in size by *n*, the algorithmic complexity is bounded by *O*(*n* ^2^), where *n* is the number of character states. Across 1,000 runtime comparisons, where 50 tip states were randomly generated for a six‐state ordered character, this was found to result in a fivefold speed increase in the calculation of *g* relative to explicit, tree‐based optimization using generalized parsimony *sensu* Swofford and Maddison (1992) on the star tree.

### Maximum possible cost (*g*_max_) among general cost matrix characters

#### General algorithm 3: exhaustive calculation of maximum possible cost (*g*_max_)

In general, exhaustive calculation of *g*_max_ can be performed by combinatorial generation of all possible integer frequencies of *n* states among *t* taxa, with restrictions (i)–(iii) above (such that *t* and *n* are met). This is implemented in Claddis by the permute_restricted_compositions function, which in turn uses the multicool (Curran et al., 2021) and partitions (Hankin, 2006) R packages. Algorithm 2 for the calculation of *g*, described above, can then be applied to all possible state frequency distributions, and the costs compared. The highest returned value of *g* for any possible state frequency distribution is equal to *g*_max_ (e.g. Fig. 3c). As this is an exhaustive search, this algorithm is guaranteed to find the maximal cost, *g*_max_. The problem size for this algorithm is given by the *k*‐dimensional extension of the triangular number (e.g. Baumann, 2019), which in our case (i.e. meeting conditions (i)–(iii)) is the binomial coefficient $\left(\begin{array}{c} t-1\\ n-1 \end{array}\right)$. Expressed in big‐*O* notation, the complexity of general algorithm 3, for *g*_max_, is then given by the complexity order for this binomial, multiplied by the complexity of algorithm 2 for *g* (above), giving an upper bound on complexity of order *O*(*t* ^(*t*−1)^ *n* ^2^) = *O*(*t* ^(*n*−1)^ *n* ^2^).

### Algebra‐based calculation of maximum possible cost

In general for a cost matrix character (see Appendix A for a full list of applicable character types, and simplifications and modifications where relevant), with *t* taxa, *n* states and given transition costs, mathematical models of the most costly state frequencies and corresponding maximum parsimony cost can be constructed that make use of linear equations, specified in terms of the number of taxa and costs in the specified cost matrix, as follows. The initial algebraic argument below is not constrained to meet conditions (i)–(iii) (i.e. state frequencies may be theoretically any real number). In the following section (methods of calculating maximum parsimony cost: comparisons and interpretation), we then show how this algebraic argument can be developed to apply conditions (i)–(iii) and calculate *g*_max_ (as defined, above, to meet conditions (i)–(iii)).

#### Algebraic derivation of maximum possible cost in binary characters

The derivation of algebraic models for state frequencies, and correspondingly maximum cost, is based on the solution of simultaneous linear equations for the implied cost if each state is the ancestral state for all state transitions (i.e. if a state is ancestral on the star tree). In general, finding maximum cost on the star tree can be considered as an optimization problem in which the aim is to maximize, across all possible state frequency combinations, the minimum cost if any one of the *n* given states for *t* taxa is ancestral. This type of optimization problem, in which maximization of minima is considered, is sometimes called a maximin problem in linear programming.

Proof 2 Here we propose, and provide an algebraic proof of, a general formula for maximum cost for binary characters. For binary characters (subject to the rules defining a valid cladistic character above), where the implied ancestral costs for both states are equal, the frequency of one state could not be increased without making the other state more parsimoniously ancestral. Therefore, at this point, cost is maximized.

For a binary character with two states (here numbered states 0 and 1, according to increasing order in their specified cost matrix), given a frequency of state 0 (denoted *x*), the frequency of state 1 (denoted *y*) is automatically.

(1) y=t–x

Correspondingly,

(2) x=t–y

If state 0 is the only ancestral state (on a star tree or conceptual equivalent), the implied total transition cost (denoted *u*_0_) will be equal to the frequency of the other state, state 1, times the cost of a transition from state 0 to 1 (*c*_0,1_).

Therefore,

(3) $$u_{0}=c_{0,1}\left(t-x\right)$$

Conversely, if state 1 is the only ancestral state the implied total transition cost (denoted *u*_1_) will be equal to the frequency of the other state, state 0, times the cost of a transition from state 1 to 0 (*c*_1,0_).

Therefore,

(4) $$u_{1}=c_{1,0}\left(t-y\right)$$

If these costs are equal,

(5) $$u_{0}=u_{1}$$

and the left‐hand sides of eqs (3) and (4) are equal; therefore the right‐hand sides are also equal.

Consequently,

(6) $$c_{0,1}\left(t-x\right)=c_{1,0}\left(t-y\right)$$

Equation (6) can then be rearranged to give the following formula for *y* (the frequency of state 1), when the costs of either state being ancestral are equal.

(7) $$y=\frac{c_{0,1}x-c_{0,1}t}{c_{1,0}}+t$$

The right‐hand side of eq. (1) can be substituted for the left‐hand side of eq. (7) to eliminate *y* as a variable.

(8) $$t-x=\frac{c_{0,1}x-c_{0,1}t}{c_{1,0}}+t$$

Equation (8) can then be solved for *x* to give the following formula for the frequency of state 0, when the costs of either state being ancestral are equal.

(9) $$x=t\frac{c_{0,1}}{c_{0,1}+c_{1,0}}$$

Where the implied transition costs are equal if either of two states is ancestral, the corresponding cost can be calculated by considering either of the two possible ancestral states (as consideration of either will necessarily give the same result). Considering state 1 as ancestral, cost will be given by the frequency of state 0 (*x*) times the cost of transition from state 1 to state 0. Substituting in the right‐hand side of eq. (9) for *x* and rearranging gives the following formula for maximum cost under maximum parsimony, where the costs if either state is ancestral are equal:

(10) $$u_{0}=u_{1}=t\frac{c_{0,1}c_{1,0}}{c_{0,1}+c_{1,0}}$$

where *t* is the number of taxa, *c*_0,1_ is the cost of transition from state 0 to state 1, and *c*_1,0_ is the transition cost from state 1 to state 0. This completes the proof.

#### Special case: binary characters with transition cost 1 between different states

For unordered characters, with transition cost 1 for either forward transitions (i.e. gains e.g. transitions from state 0 to state 1) or backward transitions (i.e. losses e.g. transitions from state 1 to state 0), it has been proved graph theoretically (Hoyal Cuthill et al., 2010), by connection to Erdös–Székely path systems (Erdös and Székely, 1992; Semple and Steel, 2003), that maximum character steps are given by the maximum frequency of a least frequent state, which can be calculated as *g*_max_(*t*, *n*) = *t* − ⌈*t*/*n*⌉, as introduced above. So, for binary unordered characters, *g*_max_(*t*, 2) = *t* − ⌈*t*/2⌉. For integer values of *t*, this is equivalent to *g*_max_(*t*, 2) = ⌊*t* − *t*/2⌋ = ⌊*t*/2⌋, where the floor operation returns the largest integer less than or equal to *t*/2.

If transition costs between states are 1, then the algebraic formula above for maximum cost in binary cost matrix characters (eq. (10)) also simplifies to  t/2. Equation (10) above is applicable to both general binary cost matrix characters and binary characters with costs for transition between different states restricted to 1. Therefore, calculation of *g*_max_ in the latter case (Hoyal Cuthill et al., 2010) is a special case of the general formula for maximum parsimony cost in binary cost matrix characters presented here (eq. (10)).(eq. (10)).

#### Algebraic derivation of formulae for cost in multistate characters

Proof 3 Here we propose and give an algebraic proof of a general formula for cost when all ancestral state costs are equal for multistate characters.

A similar approach to that outlined above for binary characters can be applied to multistate cost matrix characters to derive algebraic models of state frequencies, and the corresponding cost, by solution of simultaneous equations for the implied cost if each state is ancestral for all transitions.

For instance, for a three‐state character, the frequency (*x*) of the first state (denoted state 0) in a specified cost matrix is given by the number of taxa minus the sum of the frequency (*y*) of the second (state 1) and the frequency (*z*) of the third state (state 2):

(11) x=t−y−z

Similarly,

(12) y=t−x−z

(13) z=t−x−y

If state 0 is the only ancestral state, the implied total transition cost (*u*_0_) will be given by the frequencies of the other two states (states 1 and 2) times their production costs, the respective costs of a transition from state 0 to 1 (*c*_0,1_) or from 0 to 2 (*c*_0,2_).

Therefore,

(14) $$u_{0}=c_{0,1}\left(t-x-z\right)+c_{0,2}\left(t-x-y\right)$$

Similarly, the implied cost (*u*_*1*_) if state 1 is ancestral is

(15) $$u_{1}=c_{1,0}\left(t-y-z\right)+c_{1,2}\left(t-x-y\right)$$

and the implied cost (*u*_*2*_) if state 2 is ancestral is

(16) $$u_{2}=c_{2,0}\left(t-y-z\right)+c_{2,1}\left(t-x-z\right)$$

If these costs are equal, then

(17) $$u_{0}=u_{1}=u_{2}$$

The equations above then provide the following system of linear equations:

(11) x=t−y−z

(12) y=t−x−z

(13) z=t−x−y

(18) $$c_{0,1}\left(t-x-z\right)+c_{0,2}\left(t-x-y\right)=c_{1,0}\left(t-y-z\right)+c_{1,2}\left(t-x-y\right)$$

(19) $$c_{0,1}\left(t-x-z\right)+c_{0,2}\left(t-x-y\right)=c_{2,0}\left(t-y-z\right)+c_{2,1}\left(t-x-z\right)$$

(20) $$c_{1,0}\left(t-y-z\right)+c_{1,2}\left(t-x-y\right)=c_{2,0}\left(t-y-z\right)+c_{2,1}\left(t-x-z\right)$$

The above system of equations can then be solved to give the following algebraic models for the frequencies (*x*, *y*, *z*) of the states (respectively states 0, 1, 2) when all ancestral costs are equal, in terms of the number of taxa (*t*) and specified transition costs. Each formula is in the form of the number of taxa multiplied by a fraction that relates the different transition costs in the cost matrix to each other.

(21) $$x=t\frac{c_{0,2}c_{2,1}-c_{1,2}\left(c_{2,1}-c_{0,1}\right)}{c_{1,0}c_{0,2}+c_{2,1}\left(c_{1,0}+c_{0,2}\right)-c_{0,1}c_{1,0}-c_{1,2}\left(c_{2,1}-c_{2,0}-c_{0,1}\right)-c_{2,0}\left(c_{0,2}-c_{0,1}\right)}$$

(22) $$y=t\frac{c_{0,2}c_{1,0}+c_{1,2}c_{2,0}-c_{0,2}c_{2,0}}{c_{1,0}c_{0,2}+c_{2,1}\left(c_{1,0}+c_{0,2}\right)-c_{0,1}c_{1,0}-c_{1,2}\left(c_{2,1}-c_{2,0}-c_{0,1}\right)-c_{2,0}\left(c_{0,2}-c_{0,1}\right)}$$

(23) $$z=t\frac{c_{0,1}c_{2,0}+c_{1,0}c_{2,1}-c_{0,1}c_{1,0}}{c_{1,0}c_{0,2}+c_{2,1}\left(c_{1,0}+c_{0,2}\right)-c_{0,1}c_{1,0}-c_{1,2}\left(c_{2,1}-c_{2,0}-c_{0,1}\right)-c_{2,0}\left(c_{0,2}-c_{0,1}\right)}$$

If state 2, for instance, is considered as ancestral (any state can be considered with equal cost), maximum cost under maximum parsimony will be given by the frequencies of the other two states times their respective production costs:

(24) $$u_{0}=u_{1}=u_{2}=xc_{2,0}+yc_{2,1}$$

Equation (24) above can then be grouped with formulae (21) and (22), for *x* and *y* respectively, giving a system of equations that can be solved to give an algebraic model for cost, when all ancestral costs are equal, in terms of the number of taxa and specified transition costs:

(25) $$\begin{array}{rcl} u_{0} & = & u_{1}=u_{2}\\ & = & t\frac{c_{0,1}c_{1,2}c_{2,0}+c_{0,2}c_{1,0}c_{2,1}}{c_{1,0}c_{0,2}+c_{2,1}\left(c_{1,0}+c_{0,2}\right)-c_{0,1}c_{1,0}-c_{1,2}\left(c_{2,1}-c_{2,0}-c_{0,1}\right)-c_{2,0}\left(c_{0,2}-c_{0,1}\right)} \end{array}$$

This completes the proof.

Algebraic equation systems and resultant formulae for costs, when all ancestral costs are equal, and corresponding state frequencies for four‐state characters, such as DNA and RNA, are provided as Supplementary Information (owing to the length of the formulae). The equation systems and solutions described above for binary and three‐state characters are also provided in the Supplementary Information with single character variable names (e.g. for ease of use in computer code), in addition to their statement in the text above.

#### Special case: unordered three‐state characters

Similarly to binary characters with off‐diagonal costs equal to 1, above, among unordered characters which have all transition costs between different states equal to 1, the formula *g*_max_(*t*, *n*) = *t* − ⌈*t*/*n*⌉ (Hoyal Cuthill et al., 2010) gives for characters with three states *g*_max_(*t*, 3) = *t* − ⌈*t*/3⌉. With integer values of *t*, this is equivalent to *g*_max_(*t*, 3) = ⌊*t* − *t*/3⌋, which simplifies to $g_{\max}=\left\lfloor \frac{2}{3}t\right\rfloor$ (Steel and Charleston, 1995; Goloboff and Wilkinson, 2018). Likewise, if all transitions between different states have cost 1, the formula for cost when all ancestral costs are equal in three‐state cost matrix characters above (eq. (25)) simplifies, when *n* = 3, to $u_{0}=u_{1}=u_{2}=\frac{2}{3}t$. Therefore, the measurement of maximum possible cost among unordered three‐state characters is again shown to be a special case of the cost where all ancestral costs are equal for general cost matrix characters.

### General algorithm 4: algorithm for linear algebraic calculation of equal ancestral costs for general cost matrix characters

#### Pseudocode description

The algorithm finds the character state frequencies where the implied costs are equal if any given state is ancestral (e.g. eq. (5) and eq. (17), above) and returns the corresponding cost, using programmatic construction and solution of appropriate matrices representing systems of linear equations. A system of *n* linear equations can be represented as a matrix of coefficients of the *n* unknowns, with one row for the coefficients of each equation and one column for each unknown (here representing the frequency *x*, *y*, *z*, … of each state 0, 1, 2, …, *n* − 1), accompanied by a column matrix of the *n* constants to which the left‐hand sides of each equation (giving the sum of unknowns times their coefficients) are equal. When such matrices have been constructed, automated linear algebra can then be used to return the values of the unknowns which satisfy the system of equations, for any number of states (within computational limits). We construct the required matrices as follows:

1. The first row in the coefficient matrix is filled with ones and the first row in the constant matrix is filled with the number of taxa *t*. By this, the first rows of the coefficient and constant matrices represent the fact that the frequencies of the states (each with coefficient 1) sum to the number of taxa (e.g. see eqs (1) and (11) above for 2 and 3 states, respectively). For example, for three states, the following equation:
1x+1y+1z=t
  is represented in matrix form as
$$\left(1\;1\;1\right)\left(\begin{array}{c} x\\ y\\ z \end{array}\right)=\left(t\right)$$
  where the left matrix is the coefficient matrix and the right matrix is the constant matrix.
2. The remaining *n* − 1 rows of the coefficient and constant matrices are filled as follows. For each character state *i* = 2, …, *n*, take the *i*th row in the cost matrix and subtract this from the first row in the cost matrix. Enter the resultant difference as the *i*th row of the coefficient matrix. Enter 0 in the *i*th row of the constant matrix. The *i*th row of the coefficient and cost matrix together represent an equation equating the implied cost if the first state is ancestral to the implied cost if the *i*th state is ancestral (e.g. eqs (6) and (18)). For example, we can take the following three‐state cost matrix:
$$\left(\begin{array}{ccc} c_{0,0} & c_{0,1} & c_{0,2}\\ c_{1,0} & c_{1,1} & c_{1,2}\\ c_{2,0} & c_{2,1} & c_{2,2} \end{array}\right)$$
  If the cost $u_{0}$, if state 0 is ancestral, is equal to the cost $u_{1}$, if state 1 is ancestral, then $u_{0}=u_{1}$, so
$$c_{0,0}x+c_{0,1}y+c_{0,2}z=c_{1,0}x+c_{1,1}y+c_{1,2}z$$
  - by eq. (18) above. This equation can be rewritten by subtracting the right‐hand‐side from the left‐hand‐side and writing the left‐hand‐side of the result, in matrix form, into the second row of the coefficient matrix, and the right‐hand‐side of the result into the second row of the constant matrix. A similar process conducted for the first and third states is used to fill the third rows of the coefficient and constant matrices. This produces the following rewriting of the system of equations in matrix form:
$$\left(\begin{array}{ccc} 1 & 1 & 1\\ c_{0,0}-c_{1,0} & c_{0,1}-c_{1,1} & c_{0,2}-c_{1,2}\\ c_{0,0}-c_{2,0} & c_{0,1}-c_{2,1} & c_{0,2}-c_{2,2} \end{array}\right)\left(\begin{array}{c} x\\ y\\ z \end{array}\right)=\left(\begin{array}{c} t\\ 0\\ 0 \end{array}\right)$$
  When the cost if each state *i* = 2, …, *n* is ancestral has been equated to the cost if the first state (*i* = 1) is ancestral, the costs if any given state is ancestral have been equated by transitivity. Therefore, the system of *n* linear equations represented by the coefficient and constant matrices can then be solved to return the coefficients of the *n* state frequencies that satisfy these equations.
3. The implied number of parsimony steps if the last state, number *n*, is ancestral is then calculated (although the algorithm could be adjusted to use any state with equivalent result, as described above). The last row of the cost matrix, representing the costs of generating one of each other state in the cost matrix from the last state, is multiplied by the state frequencies returned in step 2. These values are then summed to give the total cost of generating all states, at their calculated frequencies, if the last state is ancestral (e.g. eqs (9) and (25), above).

The algorithmic complexity of solving a system of *n* linear equations is at most *O*(*n* ^3^) (Pan, 1991).

### Methods of calculating maximum parsimony cost: comparisons and interpretation

Algebraic and algorithmic maximization of most parsimonious cost on the star tree (as outlined above) provides an explanation of when and why maximum parsimony cost, and therefore maxima of many measures of homoplasy (as introduced above), are maximized among cladistic characters in general (across general cost matrix characters, including, for example, ordered and unordered characters). The potential for homoplasy is maximized by distributions of character state frequencies among taxa that imply the highest possible real‐number cost that could be achieved, where no other ancestral state gives lower cost with the same state frequencies. The application of further constraints ((i)–(iii) above) to model the majority of cladistic characters, with state frequencies meeting specified natural numbers of taxa and states, requires the further determination of the maximum cost (where there is no cheaper ancestral state) that is achievable given these conditions. As described above, it is this maximal cost value which we here denote *g*_max_, for comparability with real cladistic characters.

For binary characters, the most costly real‐number state frequencies are achieved when state frequencies among *t* taxa are maximized given their relative production costs such that ancestral costs are equal for both states (e.g. Fig. 3c).

**Fig. 4:**
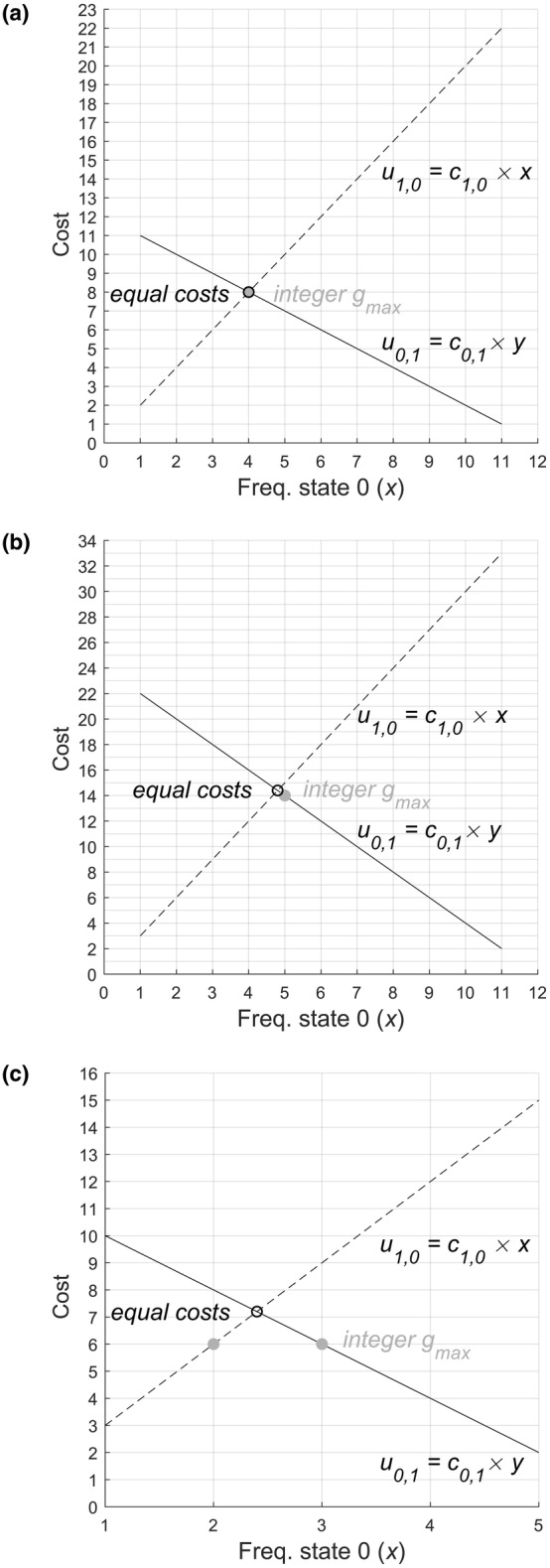
Binary cost matrix examples comparing the exhaustively calculated value of *g*_max_ for simulated integer state frequencies, where *t* is met and *n* is met (grey filled circles) with the algebraic value where ancestral costs are equal (from eq. (10)) (black open circles). The grid indicates integer values meeting *t* and *n*. (a) Example where equal costs are achieved by integer state frequencies and equal *g*_max_. Cost matrix (top left to bottom right): (0, 1; 2, 0). Number of taxa *t* = 12. (b) Example where equal costs and corresponding state frequencies are non‐integer and *g*_max_ is the closest implied cost that can be achieved by integer state frequencies. Cost matrix: (0, 2; 3, 0), *t* = 12. (c) Example where there are two combinations of integer state frequencies which are as close as possible to the equal cost. Cost matrix: (0, 2; 3, 0), *t* = 6.

Proof 4 Proof of the proposition that cost is maximized for general binary characters when ancestral state costs are equal. It can be seen this is the case for general two‐state characters, as follows. Since negative transition costs are prohibited (Rule 3) and the frequencies of the states sum to the number of taxa, an increase in the frequency of one state must lead to increase in the implied ancestral cost if the other state is ancestral. Consequently, the slopes relating ancestral cost and first state frequency must have opposite sign (e.g. Fig. 4), with the ancestral cost for the first state negatively linearly related to the frequency of the first state (by eq. (3)) and the ancestral cost for the second state positively linearly related to the frequency of the first state (by eq. (4)). Consequently, cost is maximized at the point of equal ancestral costs where a further increase in the frequency of a state would make the ancestral cost of that state cheaper and the ancestral cost of the other state more expensive (such that the cheaper state would be most parsimoniously ancestral). This completes the proof.

Proof 5 Proof 4 now permits the proposition and proof, for general binary characters, of a function (eq. (26)) for the maximal cost *g*_max_ achievable with integer state frequencies that meet *t* and *n* (meeting conditions (i)–(iii)), as follows. Where ancestral costs are equal (eq. (5)), eq. (9), derived above, gives the corresponding frequency *x* of the first state. This frequency *x* can then be subtracted from the number of taxa, *t*, to give the corresponding frequency *y* of the second state (eq. (1)). Given an integer value of *t*, if frequency *x* is an integer, frequency *y* must also be integer. Given Proof 4, above, for a binary character, the cost where both ancestral costs are equal must either be achievable with an integer frequency *x* of the first state, in which case it is *g*_max_ (e.g. Fig. 4a) or the value of *x* corresponding to equal costs must lie between two possible integer values of *x* (e.g. Fig. 4b,c). In either case, condition (i) is met. The maximum integer achievable cost, *g*_max_, where no state can be ancestral with cheaper cost, is then given by the maximum, for these two possible integer values of frequency *x*, of the minimum cost if either state is ancestral. For binary general cost matrix characters, therefore:

(26) $$g_{\max}=\max\left(\begin{array}{c} \min\left(\begin{array}{c} \left(t-\left\lfloor t\frac{c_{0,1}}{c_{0,1}+c_{1,0}}\right\rfloor \right)c_{0,1},\\ \left\lfloor t\frac{c_{0,1}}{c_{0,1}+c_{1,0}}\right\rfloor c_{1,0} \end{array}\right),\\ \min\left(\begin{array}{c} \left(t-\left\lceil t\frac{c_{0,1}}{c_{0,1}+c_{1,0}}\right\rceil \right)c_{0,1},\\ \left\lceil t\frac{c_{0,1}}{c_{0,1}+c_{1,0}}\right\rceil c_{1,0} \end{array}\right) \end{array}\right)$$

It can be seen that the value of *g*_max_ returned by eq. (26) meets conditions (i)–(iii), as follows. By Rule 3 for a valid cost matrix (Fig. 2), off‐diagonal costs must be positive (*c*_0,1_ ≥ 1, *c*_1,0_ ≥ 1). If neither *c*_0,1_ nor *c*_1,0_ is negative or 0, by eq. (9) the frequency *x* of state 0 cannot be negative or 0. Therefore, *x* is greater than zero and the ceiling of *x* (eq. (26)) is at least 1. If the ceiling of *x* is 1, the minimum ancestral cost given ceiling *x* will be greater than that given floor *x*, which implies a disallowed zero *x*, and the disallowed ancestral cost will not be the maximum returned as *g*_max_. Similarly, by eq. (9), given *c*_0,1_ ≥ 1, *c*_1,0_ ≥ 1, *x* must be less than *t*. If the ceiling on *x* is equal to *t*, which (by eq. 26) implies a disallowed frequency *y* of zero, the floor of *x* will be *t* − 1, the corresponding value of *y* will be at least 1 and the corresponding value of *g*_max_ will be selected over the disallowed cost. Consequently, the value of *g*_max_ returned by eq. (26) meets the required conditions (i)–(iii), completing the proof.

Since costs if each state is ancestral are linearly related to the frequency *x* of the first state, and the cost where costs are equal is maximal (Proof 4), for binary general cost matrix characters, *g*_max_ must, therefore, be the highest cost, achievable with integer state frequencies between 1 and *t* − 1, that is less than or equal to equal ancestral costs.

Interestingly, where off‐diagonal transition costs differ (e.g. in a binary asymmetric character, Fig. 3c) the most costly state frequency distributions are not necessarily the most balanced possible distribution of states among taxa (as in unordered characters, Hoyal Cuthill et al., 2010) but are rather distributions of taxa among states which reflect relative state production costs and may therefore show unequal state frequencies (e.g. Figs 3c and 4).

For unordered multistate characters (with *n* ≥ 2 and off‐diagonal costs of 1) it can be proved that the cost when ancestral costs are equal for all states is equal to the maximal real number cost, with the closest integer value previously proved to be a maximum bound on most parsimonious steps *s* for unordered characters (Hoyal Cuthill et al., 2010), as follows.

Proof 6 Here we prove this proposition that, for general unordered characters, t‐t/n is equivalent to the cost when ancestral costs are equal (calculated by general algorithm 4), by showing that both are the costs where state frequencies are equal. Maximal parsimony cost t‐t/n is equal to the cost t−F, where *F* is the frequency of a most frequent character state (Hoyal Cuthill et al., 2010) that is not constrained by conditions (i)–(iii) to be necessarily an integer between 1 and *t* − (*n* − 1). t−F is maximized when character state frequencies are equal (Mickevich, 1978; Steel and Penny, 2006; Hoyal Cuthill et al., 2010). When ancestral costs on the star tree are equal for all states for an unordered character (as determined by general algorithm 4), the sum of costs of producing all states multiplied by the frequencies of the states is equal, whichever state is considered as ancestral. Since, for an unordered character where diagonal costs are zero and all other costs are 1, the cost of producing all other states from any ancestral state is given by their respective frequencies (which are all multiplied by the cost of 1). Therefore, since ancestral costs are equal for all states, state frequencies must also be equal. Therefore, for unordered characters, t‐t/n is also the cost when ancestral costs are equal for all states, completing the proof.

**Fig. 5:**
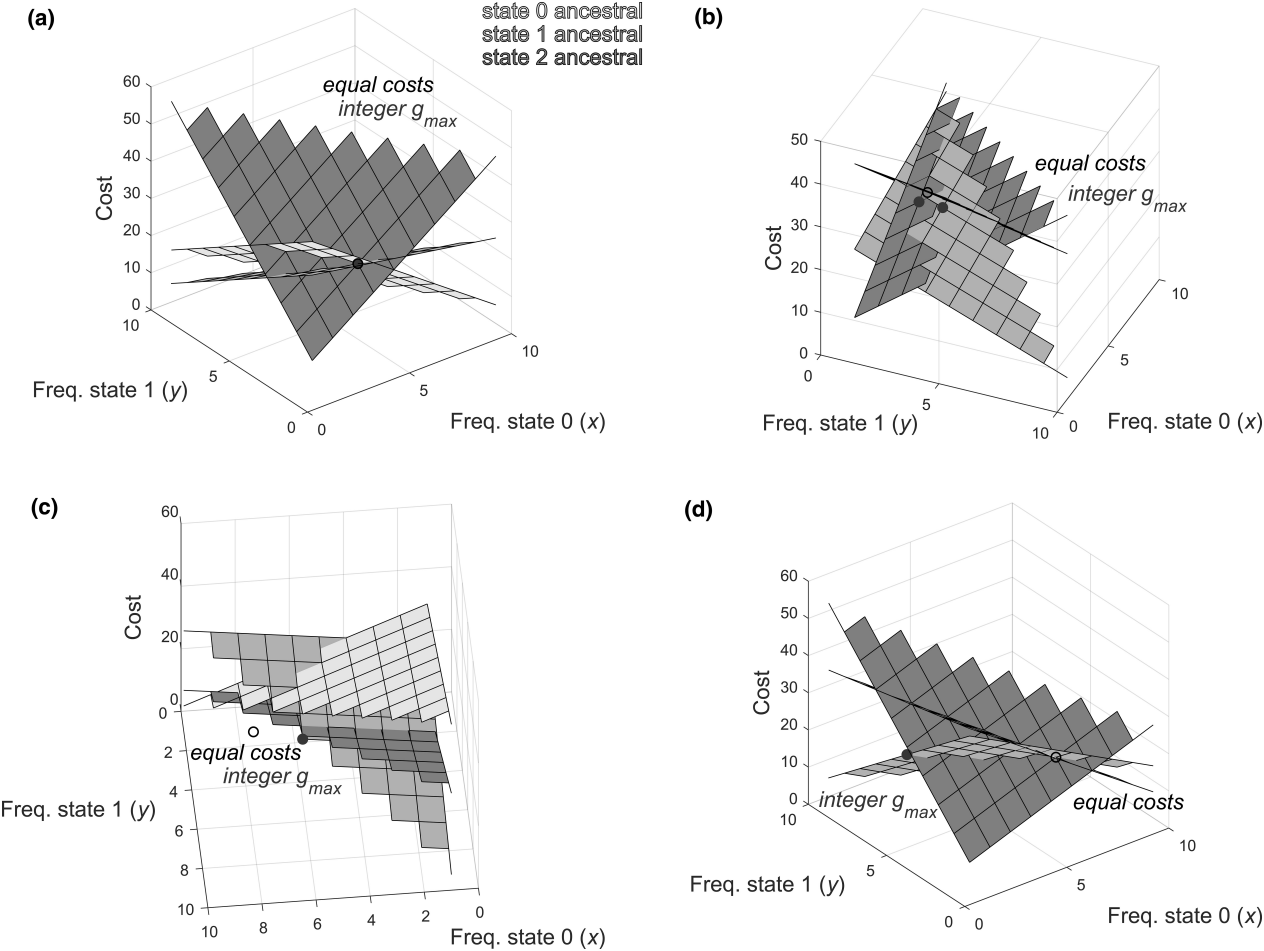
Three‐state cost matrix examples comparing the exhaustively calculated value of *g*_max_ across simulated integer state frequencies between 1 and *t* − (*n* − 1) (meeting conditions (i)–(iii); grey filled circles) with the algebraic maximal cost when all ancestral costs are equal (eq. (25)) (black open circles). Each of the three planes shows the implied cost (vertical axis) if that state is ancestral (non‐italic key), for each possible combination of state frequencies that sums to give the number of taxa (*t*). Grids indicate the integer values where *t* and *n* are met. (a) Example where equal costs are achievable with these integer state frequencies and equal the exhaustive value (labelled integer *g*_max_). Cost matrix (top left to bottom right): (0, 1, 4; 2, 0, 3; 4, 5, 0), *t* = 12. (b) Example where the exhaustive value of *g*_max_ that can be achieved with state values meeting conditions (i)–(iii) is as close as possible to, but not equal to, algebraic equal costs. Cost matrix: (0, 3, 4; 3, 0, 2; 3, 4, 0), *t* = 12. (c) Example where the planes representing implied costs intersect outside the allowed state space, because the equal cost frequency of state 2 is negative, and *g*_max_ is at the boundary ($u_{0}=u_{1}=u_{2}=22.2$: *t* = *x* + *y* + *z* = 12, *x* = 7.8, *y* = 4.8, *z* = −0.6; *g*_max_ = 20: *x* = 6, *y* = 5, *z* = 1). Cost matrix: (0, 5, 3; 3, 0, 2; 1, 3, 0). (d) Example where the planes representing implied costs intersect inside the allowed state space but *g*_max_ is higher than equal ancestral costs and is at the boundary of the state space ($u_{0}=u_{1}=u_{2}=22$: *t* = *x* + *y* + *z* = 12, *x* = 6, *y* = 2, *z* = 4; *g*_max_ = 25: *x* = 1, *y* = 5, *z* = 6). Cost matrix: (0, 3, 4; 1, 0, 4; 2, 5, 0).

For general cost matrix characters (where costs of transitions between different states can deviate from 1) that have more than two states, however, we demonstrate by example (Fig. 5d) that there are cases in which maximal cost (an ancestral cost where no alternative state can be selected as the ancestor with cheaper cost) does not occur at the point of equal ancestral costs for all states. In examples, for some custom cost matrices permitted by the rules outlined here (e.g. Fig. 5d), the maximal integer‐achievable cost meeting conditions (i)–(iii) is instead achieved in the region of a boundary of the allowed state space.

With some specific modifications, in particular, for irreversible and Dollo characters (see Appendix A), the general framework (above) permits (with the provisos detailed) the calculation of potential evolutionary ranges (*m* to *g*_max_) under maximum parsimony across all discrete character types observed among 4,467 character matrices (as specified in Appendix A). The exhaustive algorithms for *m* (1), *g* (2) and *g*_max_ (3) outlined above provide general solutions. However, the algorithmic complexity of the exhaustive search for *g*_max_, which searches across all allowed character state frequency combinations, is particularly notable as dependent on both the number of taxa (*t*) and character states (*n*) (*O*(*t* ^(*n*−1)^ *n* ^2^), above). Consequently, standard computational limits are likely to make exhaustive calculation of *g*_max_ infeasible for high numbers of taxa and/or states. This makes applicable formulae for the calculation of *m*, *g* or *g*_max_ with reduced (particularly constant) run‐time requirements with respect to problem size (particularly *t* or *n*) valuable wherever possible. In Table 1 and Appendix A we summarize the known and new formulae for each character type, where available. Character types covered by specific formulae include the most commonly used types of binary symmetric (used in 100% of surveyed datasets), multistate unordered symmetric (89.8%) and multistate linear ordered (47.6%), as well as a range of more rarely used (but still biologically important) characters, including Dollo (<0.1%) and irreversible characters (binary 0.6%, multistate 0.5%).

## Conflict of interest

The authors declare no conflict of interest.

## Supporting information


**Data S1.** Supporting Information.

## Figures and Tables

**Fig. A1:**
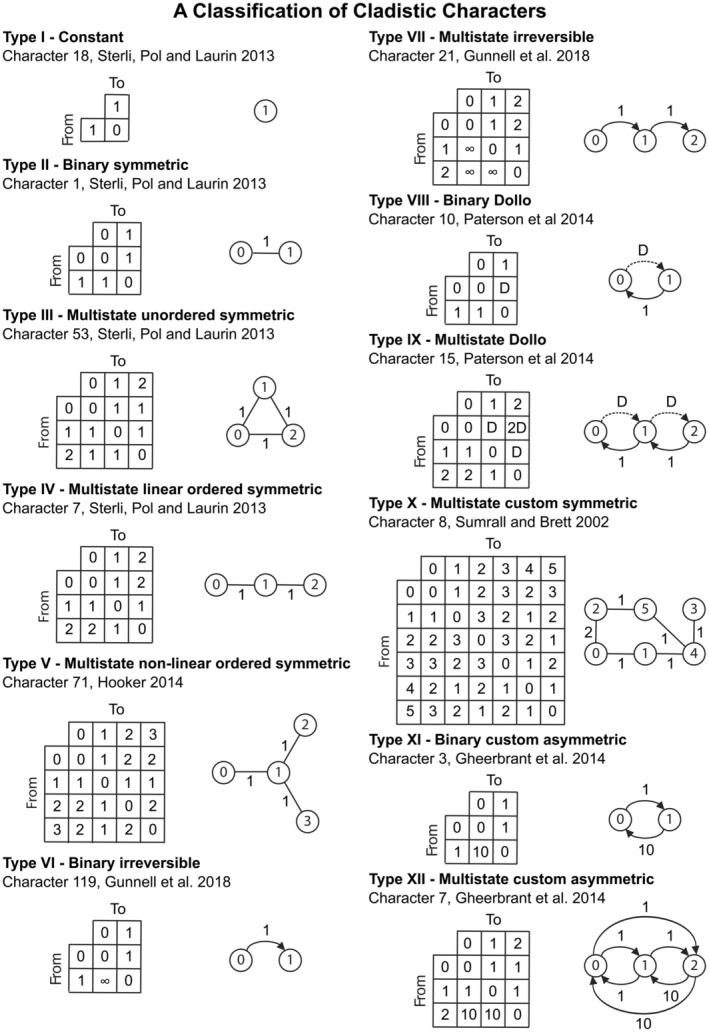
A classification of cladistic characters into 12 types with an empirical example for each shown as both a cost matrix and state (di)graph. For Type VIII and Type IX Dollo characters, the *D* term is a finite number that penalizes particular transitions (shown as dashed arcs in the state digraph) such that they will only be traversed once (Swofford and Olsen, 1990). In Table 1 and Appendix A we provide and prove specific values for *D* as described in Appendix A.

## Data Availability

Published cladistic data matrices surveyed in this study are available via https://www.graemetlloyd.com.

## Appendix A

### A classification of cladistic characters and specification of methods of calculation of minimum (*m*), maximum (*g*) and maximum possible (*g*_max_) costs under maximum parsimony

Cladistic characters come in a wide variety of forms. Here we classify these into 12 distinct types (Fig. A1) and provide definitions for each, as both cost matrices and state (di)graphs. Below, we provide further details on how *m*, *g* and *g*_max_ may be calculated for each type and specify relevant published or provided proof(s) for each case. Calculation of *m*, *g* and *g*_max_ can be performed for many distinguishable types using the general solutions outlined above for general cost matrix characters. For character subtypes for which these general solutions require specific modifications, or where specific simplifications are of interest, we describe or provide individual solutions. A general algorithm (Sankoff, 1975; Sankoff and Rousseau, 1975; Sankoff and Cedergren, 1983) for calculating *s* for Types I–VII and X–XII was already described in Swofford and Maddison (1992) and a specific algorithm for Types VIII and IX by Swofford and Olsen (1990); *s* is not discussed further here. We state whether a character type can be coded using the NEXUS format (Maddison et al., 1997), used by PAUP* and Claddis, or TNT format and whether implementations for *m*, *g* and *g*_max_ are available in Claddis and the maximum parsimony software PAUP* and TNT. We also note the frequency of each character type in the literature (based on 4,467 cladistic datasets representing an updated version of the database of Wright et al., 2016 and Wright and Lloyd, 2020). A single example for each type is shown in Fig. A1. Claddis can automatically treat character types based on an input cost matrix specified in NEXUS format (using the classify_costmatrix function).

As introduced above, unless otherwise stated for a given character type, we assume that any state may be potentially ancestral and values such as *m* and *g*_max_ are then optimized accordingly given the cost matrix and number of taxa. However, we note that it may be possible to modify a number of the algorithms and formulae we provide for cases in which additional constraints are specified, for example where an outgroup state may be forced to be the ancestral root state (as in the TNT and PAUP* ancstates commands).

### Type I—constant characters

Definition: an invariant or constant character, where all coded (i.e. not missing and not inapplicable) states are identical. There is only a single transition cost, *c*_0,0_ = 0.

State graph: a single vertex with no loops, equivalent to both the complete graph *k*_1_ or the path graph *P*_1_.

NEXUS/TNT coding: various codings (see Type II–XII characters below) could be used for constant characters as no transitions can occur.

Empirical frequency: although such characters are parsimony uninformative they occur commonly in empirical data, with at least one such character found in 1,351 of 4,467 (30.2%) datasets. For an example of usage see character 18 in Sterli et al. (2013).

Calculating *m*: under character Rule 2 any transition cost *c*_*i,j*_, where *i* = *j*, must be zero, therefore *m* = 0 (as it is not possible to infer any changes). This character type is also covered by the general solution for *m* based on the length of the minimum spanning tree (see “General algorithm 1: minimum cost, *m* for cost matrix characters”, above).

Calculating *g*: as no changes are permitted under parsimony for a constant character, it follows that *g* = 0. This character type is also covered by the general cost matrix character solution for the algorithmic calculation of *g* (see “General algorithm 2: maximum cost (*g*) among general cost matrix characters”, above).

Calculating *g*_max_: as all tips can only be assigned to a single state, it follows that *g*_max_ = 0.

Proof: for *m* see Proof 1, above. For *g* and *g*_max_: as there is only one state the cost matrix *must* be of dimension one‐by‐one, and hence there is no off‐diagonal and so Rule 3 does not apply (there are no positive transition costs). Thus, under Rule 2 all transitions must be cost zero and by extension so must the length of any most parsimonious tree. This character type is also covered by “General algorithm 3: exhaustive calculation of maximum possible cost (*g*_max_)”, above.

Practical implementation(s): cladistic packages PAUP*, TNT and Claddis infer *m* = *g* = 0 for constant characters. Claddis additionally returns *g*_max_ = 0.

### Type II—binary symmetric characters

Definition: any binary (two state) character where *c*_0,1_ = *c*_1,0_. If *c*_0,1_ = *c*_1,0_ = 1 then we call this the “standard” binary symmetric character.

State graph: two vertices connected by a single edge and equivalent to both the complete graph *k*_2_ and the path graph *P*_2_. The single edge must have finite positive weight.

NEXUS/TNT coding: in practice a binary symmetric character may be defined as ordered (ord in NEXUS format, ccode + in TNT format) or unordered (unord in NEXUS format, ccode ‐ in TNT format). If *c*_0,1_ = *c*_1,0_ *≠* 1, then the user can define either a character state tree (usertype cstree in NEXUS format, cstree in TNT format) or cost matrix (usertype stepmatrix in NEXUS format, smatrix in TNT format) instead.

Empirical frequency: extremely common, with at least one such character found in 4,465 of 4,467 (100.0%) datasets. For example, see character 1 of Sterli et al. (2013).

Calculating *m*: *m* = *w*(*n* − 1) = *w*(2–1) = *w*, where *w* is the edge weight (i.e. the single unique non‐zero cost in the cost matrix). If the transition cost between states is 1 (*c*_0,1_ = *c*_1,0_ = *w* = 1), *m* = *n* − 1, where *n* is the number of states. Where both states of a binary character are represented (i.e. the character is not constant) *n* = 2 and *m* = 2–1 = 1. This character type is also covered by the general character state graph solution for the algorithmic calculation of *m* (general algorithm 1).

Calculating *g*: if *c*_0,1_ = *c*_1,0_ = 1, *g* = *t* − *F*, where *F* is the number of taxa with a most frequent state (Steel and Penny, 2006). If *w* = *c*_0,1_ = *c*_1,0_ ≠ 1, it is useful to apply *g* = *w*(*t* − *F*). This character type is also covered by general algorithm 2.

Calculating *g*_max_: if *c*_0,1_ = *c*_1,0_ = 1, *g*_max_ = *t* − ⌈*t*/*n*⌉, for general unordered multistate characters (Hoyal Cuthill et al., 2010), with *n* = 2, giving *g*_max_ = *t* − ⌈*t*/2⌉. If *w* = *c*_0,1_ = *c*_1,0_ ≠ 1, we apply *g*_max_ = *w*(*t* − ⌈*t*/2⌉). This character type is also covered by the general cost matrix character solutions for the algorithmic calculation of *g*_max_ with integer state frequencies (general algorithm 3, above).

Proof: for *m* see Proof 1, above, for *g* see Goloboff (2022b), for *g*_max_ see proof for general unordered multistate characters of Hoyal Cuthill et al. (2010), special case of the general algebraic solution (Proof 2: “Special case: binary characters with transition costs 1 between different states”), and Proofs 4 and 5, above.

Practical implementation(s): both standard and non‐standard *m* and *g* can be found using either the describe n/diag function of PAUP* or the minmax function of TNT, although TNT requires costs to be integers. Binary symmetric characters are covered by the general solutions described here and implemented in Claddis functions for *m* (find_costmatrix_minimum_span), *g* (calculate_g) and *g*_max_ (calculate_gmax).

### Type III—multistate unordered (symmetric) characters

Definition: a character with three or more states, where every transition is direct, symmetric and of equal cost, i.e. *c*_*i,j*_ = *c*_*j,i*_ = *w*, where *w* is a positive finite value and *i* ≠ *j*. If *w* = 1 we call this the standard multistate unordered symmetric character.

State graph: the complete graph *k*_*n*_ where *n* is ≥3 and every edge is of equal weight (*w*).

NEXUS/TNT coding: the standard multistate unordered symmetric character (*w* = 1) is representable using unord in NEXUS format or ccode ‐ in TNT format. If *w ≠* 1, then the user can define either a character state tree (usertype cstree in NEXUS format, cstree in TNT format) or cost matrix (usertype stepmatrix in NEXUS format, smatrix in TNT format) instead.

Empirical frequency: very common, with at least one such character found in 4,012 of 4,467 (89.8%) datasets. For example, see character 53 of Sterli et al. (2013).

Calculating *m*: *m* = *w*(*n* − 1), as for Type II with *n* > 2. This character type is also covered by general algorithm 1.

Calculating *g*: *g* = *w*(*t* − *F*), as for Type II with *n* > 2. This character type is also covered by general algorithm 2.

Calculating *g*_max_: *g*_max_ = *w*(*t* − ⌈*t*/*n*⌉) (Hoyal Cuthill et al., 2010) (see Type II) with *n* > 2. This character type is also covered by general algorithm 3.

Proof(s): for *m* see Proof 1, for *g* see Goloboff (2022b), and for *g*_max_ see Hoyal Cuthill et al., 2010, Proof 3: “Special case: unordered three‐state characters”, and Proof 6 above.

Practical implementation(s): both standard and non‐standard *m* and *g* can be found using either the describe n/diag function of PAUP* or the minmax function of TNT, although TNT requires costs to be integers. Type III characters are covered by the general solutions for *m*, *g* and *g*_max_ described here and implemented in Claddis.

### Type IV—multistate linear ordered (symmetric) characters

Definition: a character with three or more states where direct transitions are of equal cost and symmetric but a maximum of two direct transitions are permitted per state.

State graph: the path graph (*P*_*n*_) where *n* ≥ 3 and every edge is of equal weight, *w*.

NEXUS/TNT coding: the standard multistate linear ordered symmetric character (*w* = 1) is representable using ord in NEXUS format or ccode + in TNT format. If *w ≠* 1, then the user can define either a character state tree (usertype cstree in NEXUS format, cstree in TNT format) or cost matrix (usertype stepmatrix in NEXUS format, smatrix in TNT format) instead.

Empirical frequency: common, with at least one such character found in 2,127 of 4,467 (47.6%) datasets. For example, see Character 7 of Sterli et al. (2013).

Calculating *m*:

(A1)
$$m=c_{0,1}+c_{1,2}+\cdots +c_{n-3,n-2}+c_{n-2,n-1}=\left(n-1\right)\left(c_{0,1}\right)$$

As above, minimum steps, *m*, is equal to the length of the directed minimum spanning tree of the character state graph (general algorithm 1). Many problems in which an edge weight matrix, which is equivalently representable by a graph, must be traversed with minimum cost can be also solved using dynamic programming algorithms, which evaluate step by step the increase in cost of successive transitions through the graph or equivalent edge weight matrix (Sniedovich, 2006). For instance, Dijkstra's shortest path algorithm, which can be used to find an undirected minimum weight spanning tree through a graph, has been described both as a type of “greedy” algorithm because it selects the best choice available at each step and (without incompatibility) by a simple dynamic programming approach (Sniedovich, 2006). Dynamic programming methods have also been applied to some more complicated minimum spanning tree problems with further constraints (e.g. Nägele and Zenklusen, 2023). As introduced above, a parsimony cost matrix can be viewed equivalently as an edge weight matrix for the character state graph. Making these connections between Dijkstra's graph theoretic algorithm for the undirected minimum spanning tree, dynamic programming approaches on an edge weight matrix (Sniedovich, 2006) and a parsimony cost matrix is helpful here in the construction and proof of applicability of the formula above (eq. (A1)) for minimum steps (*m*). Dijkstra's algorithm for the undirected minimum spanning tree is applicable to symmetric cost matrices, since the length of an unrooted phylogenetic tree on such a cost matrix will not be altered by rooting because all forward and backward state transition costs are symmetric. Where Dijkstra's undirected minimum spanning tree is applicable we can consider, without loss of generality, the shortest path from a starting position of the first numbered node in an edge weight matrix and a destination of the last numbered node (Sniedovich, 2006). It follows from the above definition of a linear ordered (symmetric) character that costs in the upper off‐diagonal are each equal to $c_{0,1}$ and increase by $c_{0,1}$ in each successive position along each row (Fig. A1). Then, taking state 0 as the starting position, there must (given the stated costs) be a series of transitions, moving through the states successively, in order of state number, that adds $c_{0,1}$ at each transition (this is the path through the cost matrix along an off‐diagonal). The minimum cost *m* of traversing the *n* states in such a cost matrix will then be equal to the sum of these costs, i.e. the sum of an off‐diagonal (eq. (A1)). Since, $c_{0,1}$ is the lowest transition cost present in the cost matrix, there cannot be a path through all existing states which gives a shorter total path length than $\left(n-1\right)c_{0,1}$.

A rooted bifurcating phylogenetic tree for *t* taxa with these *n* states could be constructed from this shortest path between the states, without increase in length, by a process analogous to that outlined above for Edmond's algorithm, by attaching bifurcating subtrees each containing taxa with the same state to a node representing that state in the shortest path between states and then rooting the whole tree to any one state.

Calculating *g*: this character type is covered by general algorithm 2.

Calculating *g*_max_: this character type is covered by general algorithm 3. For a given number of character states, the constraint of symmetrical transition costs also allows corresponding simplification of the general formula for equal ancestral costs (determined by general algorithm 4). For example, for a standard three‐state linear ordered symmetric character where $c_{0,1}=c_{1,0}=c_{1,2}=c_{2,1}=1$ and $c_{0,2}=c_{2,0}=2$ (e.g. Fig. A1 Type IV) substitution of these costs into eq. (25) and simplification gives cost equal to *t*, with an unconstrained optimal frequency of the second state of zero (eq. (22)) and optimal frequencies of the first and third state equal to the number of taxa divided by two. Here, therefore, there is an optimal frequency of zero for the central state in the state graph (which has the lowest row sum in the cost matrix) and equal, maximal frequencies for the remaining two states. Similarly, for a four‐state character, substitution of costs for a Type IV character, into the equations for equal ancestral costs gives a cost of 3/2 *t*, with an unconstrained optimal frequency of the second and third states of zero and optimal frequencies of the first and third state equal to the number of taxa divided by 2. It can be seen that costs determined in this manner cannot be exceeded on the star tree, and are therefore an upper bound on maximal parsimony cost *g*_max_, because all resulting transitions must be between the existing states, which have equal, maximal inter‐state transition costs in the cost matrix and equal, maximal frequencies.

Generalizing this procedure, for an *n*‐state linear ordered, symmetric character gives a formula for maximum cost, meeting constraints (i)–(iii):

(A2)
$$g_{\max}=w\left(\left\lfloor \left(n-3\right)+\left(t-\left(n-2\right)\right)\times \left(\left(n-1\right)/2\right)\right\rfloor \right)$$

Proof(s): see argument immediately above.

Practical implementation(s): both standard and non‐standard *m* and *g* can be found using either the describe *n*/diag function of PAUP* or the minmax function of TNT, although TNT requires costs to be integers. Claddis calculates *m*, *g* and *g*_max_.

### Type V—multistate non‐linear ordered (symmetric) characters

Definition: a character with four or more states where direct transition costs are equal and symmetric and either at least one state has three or more direct transitions or every state has at least two direct transitions, without every state having a direct transition to every other state. This second feature makes a Type V character distinct from a Type IV character, and the latter makes it distinct from a Type III character.

State graph: a non‐complete connected graph of four or more vertices where either at least one vertex has degree ≥3 or every vertex has degree ≥2 (distinguishing it from a Type IV character) and all edges are of equal weight. Cycles are permitted.

NEXUS/TNT coding: the user can define a Type V character using either a character state tree (usertype cstree in NEXUS format, cstree in TNT format) or cost matrix (usertype stepmatrix in NEXUS format, smatrix in TNT format).

Empirical frequency: rare, with at least one such character found in 36 of 4,467 (0.8%) datasets. For example, see Character 71 of Hooker (2014).

Calculating *m*: as above, minimum steps, *m*, is equal to the length of the directed minimum spanning tree of the character state graph (general algorithm 1). Given this applicability of the Kruskal algorithm and equal costs of transition between different states, this character type is also covered by eq. (A1): *m* = *w*(*n* − 1).

Calculating *g*: this character type is covered by the general cost matrix character solution for the algorithmic calculation of *g* (general algorithm 2).

Calculating *g*_max_: this character type is covered by general algorithm 3.

Proof(s): see exhaustive algorithms 1–3 above.

Practical implementation(s): both standard and non‐standard *m* and *g* can be found using either the describe n/diag function of PAUP* or the minmax function of TNT, although TNT requires costs to be integers. Claddis calculates *m*, *g* and *g*_max_.

### Type VI—binary irreversible characters

Definition: a character with two states where costs are asymmetric with one cost infinite and the other finite. By convention the infinite cost is placed in the lower left triangle, but if they are placed in the upper right triangle instead the character still meets the definition of a Type VI character (see also discussion of Dollo characters below). Note: Type VI characters force both a single root state and that any homoplasy be in the form of convergent transitions. This definition is compatible with existing definitions of irreversible characters (e.g. Swofford, 2003).

State graph: a simple two‐vertex, one‐arc acyclic digraph representing a single Eulerian trail.

NEXUS/TNT coding: in NEXUS format a Type VI character can be coded as either irrev or using a stepmatrix where the lower triangle is assigned the value *i* (for infinite). In TNT Type VI characters can only be produced using the smatrix command with costs of *i* (for infinite) in the lower triangle.

Empirical frequency: rare, with at least one such character found in 27 of 4,467 (0.6%) datasets. For example, see character 119 of Gunnell et al. (2018).

Calculating *m*: we first designate the state labels such that the transition from state 1 to state 0 has infinite cost. As outlined in Proof 7 below, for an irreversible binary character, it will always be cheaper for the state which is of infinite cost to produce (here designated state 0) to be the ancestral state, so that it need not be derived within the tree. If we set the constraint that each state must be represented at least once, the minimum possible cost is then achieved if the frequency of state 1 is the minimum possible $\min\left(y\right)$, here set to be 1 (all other taxa then have state 0) and *m* is then given by the cost of producing this one instance of state 1:

(A3)
$$m=c_{0,1}$$

This character is additionally covered by general algorithm 1.

Calculating *g*: extending from the argument for *m* above,

(A4)
$$g=yc_{0,1}$$

where y is the frequency of state 1 (designating the state labels such that the transition from state 1 to state 0 has infinite cost) and the frequency x of state 0 is at least 1 (i.e. the character is genuinely binary). This character is also covered by general algorithm 2.

(A5)
$$\text{Calculating}\;g_{\max}:g_{\max}=c_{0,1}\left(t-1\right)$$

This character is also covered by general algorithm 3.

Proof(s): for *m* and *g* see above, and for *g*_max_ see Proof 7. Proof 7 Proof for the formula for *g*_max_ (proposed in eq. (A5), above) for binary irreversible characters. We will designate the state labels such that the transition from state 1 to state 0 has infinite cost: $c_{1,0}=\infty$.

By eq. (9) and Proofs 4, 5, above, the cost maximizing frequency of state 0 for a binary character is:

$$x=t\frac{c_{0,1}}{c_{0,1}+c_{1,0}}\left(9\right)$$

Denoting the cost $c_{0,1}=w$, substituting the infinite cost of reverse transition into eq. (9) gives.

(A6)
$$x=\mathrm{tw}\frac{1}{w+\infty }$$

Since one divided by a number approaches zero as that number approaches infinity, the limit on the frequency x of state 0 for an irreversible character, as $c_{1,0}$ approaches ∞, is then 0:

(A7)
$$\lim_{c_{1,0}\to \infty }x=t\frac{c_{0,1}}{c_{0,1}+c_{1,0}}=0$$

Since the frequency of the states sums to the number of taxa t (eq. (1), above), the frequency y of state 1 approaches t as $c_{1,0}$ approaches ∞:

(A8)
$$\lim_{c_{1,0}\to \infty }y=t-x=t$$

If we set a constraint that, in practice, each state must be represented at least once, then x for an irreversible binary character will therefore equal 1 and y will equal t−1, meeting conditions (i)–(iii). Under this constraint *g*_max_ is then given by the minimum of the cost if state 0 is ancestral ($u_{0}$) and the cost if state 1 is ancestral $u_{1}$.

Substituting into eqs (3) and (4) from above gives.

$$u_{0}=c_{0,1}\left(t-x\right)\left(3\right)$$

(A9)
$$u_{0}=c_{0,1}\left(t-1\right)$$

$$u_{1}=c_{1,0}\left(t-y\right)\left(4\right)$$

(A10)
$$u_{1}=\infty \left(t-\left(t-1\right)\right)$$

(A11)
$$u_{1}=\infty$$

Since the minimum of a non‐infinite number and infinity will always be the alternative to infinity, giving eq. (A12), above:

(A12)
$$g_{\max}=\min\left(u_{0},u_{1}\right)=u_{0}=c_{0,1}\left(t-1\right)$$

This completes the proof.

Practical implementation(s): both standard and non‐standard *g* can be found using either the describe n/diag function of PAUP* or the minmax function of TNT, although TNT requires costs to be integers. TNT implements *m*, with the same integer cost restriction. Claddis calculates *m*, *g* and *g*_max_.

### Type VII—multistate irreversible (asymmetric) characters

Definition: a character with three or more states where one triangle of the cost matrix is composed of infinite costs and the other triangle matches the definition of a Type IV character. By convention the infinite costs are placed in the lower left triangle, but if they are placed in the upper right triangle instead this would still meet the definition of a Type VII character. Note: Type VII characters force both a single root state and that any homoplasy be in the form of convergent transitions. This definition is compatible with existing definitions of irreversible characters (e.g. Swofford, 2003).

State graph: a simple *n*‐vertex digraph with *n* − 1 equally weighted arcs, where *n* ≥ 3 and all vertices are connected by a single Eulerian trail.

NEXUS/TNT coding: in NEXUS format a Type VII character can be coded as either irrev or using a stepmatrix where the lower triangle is assigned the value *i* (for infinite). In TNT Type VII characters can only be produced using the smatrix command with costs of *i* (for infinite) in the lower triangle.

Empirical frequency: rare, with at least one such character found in 21 of 4,467 (0.5%) datasets. For example, see character 120 of Gunnell et al. (2018). Irreversible characters also have a direct equivalent in stratigraphic congruence measures (see Bell and Lloyd 2015 and references therein).

Calculating *m*: where the upper cost matrix is of the specific form described for Type IV (eq. (A1)), above, this formula can be applied to calculate minimum steps by the sum of the upper off‐diagonal in the cost matrix (eq. (A1)). This character type is also covered by general algorithm 1.

Calculating *g*: if one state of a multistate character is the ancestral state on a star tree (or conceptual equivalent), then the total cost is given by the sum across all other states of the frequency of each state multiplied by the cost of transition from the ancestral state to the given state (e.g. for a three‐state character, eqs ((14), (15), (16)), above). We will designate state labels for a theoretical multistate irreversible character with *n* states such that states are labelled 0, 1, 2, …, *n* − 1 in order of cost matrix row indices and all infinite cost, reverse transitions are in the lower left of the matrix. We will also consider constraint (ii) that all *n* states are represented at least once among the taxa and constraint (iii) that the state frequencies sum to *t*. For such a character, no state other than state zero can be the ancestral state without requiring at least one infinite cost transition on the star tree. For example, if state 1 is ancestral, at least one instance of state 0 must be derived; however, this would imply a reverse transition from 1 to 0, which by definition for this character type has infinite cost. Therefore, as for a binary irreversible character described above (Proof 7), it will always be most parsimonious to have 0 as the ancestral state on the star tree.

Therefore, given the above, cost for a given character state distribution for an *n*‐state irreversible character will be the sum for all non‐zero states of the frequency of that state multiplied by the cost of transition to that state from 0:

(A13)
$$g=yc_{0,1}+zc_{0,2}+,\ldots ,f_{n-1}c_{0,n-1}$$

where $f_{n-1}$ is the frequency (number of copies among the taxa) of the *n* − 1th state.

Calculating *g*_max_: for a three‐state irreversible character, substituting a reverse cost denoted *i* into eq. (21) for the frequency x of state 0 and simplifying gives

(A14)
$$x=t\frac{-\left(c_{1,2}i-c_{1,2}c_{0,1}-c_{0,2}i\right)}{c_{0,1}c_{1,2}+ic_{0,2}+i^{2}}$$

Taking the limit of this eq. (A14),

(A15)
$$\lim_{i\to \infty }x=0$$

For the frequency y of state 1, substituting *i* into eq. (22) and simplifying gives:

(A16)
$$y=t\frac{ic_{1,2}}{c_{0,1}c_{1,2}+ic_{0,2}+i^{2}}$$

Taking the limit,

(A17)
$$\lim_{i\to \infty }y=0$$

For the frequency z of state two, substituting *i* into eq. (23) and simplifying gives:

(A18)
$$z=t\frac{i^{2}}{c_{0,1}c_{1,2}+ic_{0,2}+i^{2}}$$

Taking the limit,

(A19)
$$\lim_{i\to \infty }z=t$$

For *g*_max_, substituting *i* into eq. (25) and simplifying gives:

(A20)
$$u_{0}=u_{1}=u_{2}=t\frac{i\left(c_{0,1}c_{1,2}+ic_{0,2}\right)}{c_{0,1}c_{1,2}+ic_{0,2}+i^{2}}$$

Taking the limit,

(A21)
$$\lim_{i\to \infty }u_{0}=u_{1}=u_{2}=tc_{0,2}$$

If we set the constraint that each of the *n* = 3 character states must be represented at least once, then we have

(A22)
$$g_{\max}=\left(t-n+1\right)c_{0,2}+c_{0,1}$$

Generalizing this to an *n*‐state character gives

(A23)
$$g_{\max}=\left(t-n+1\right)c_{0,n-1}+c_{0,n-2},\ldots ,c_{0,1}$$

For example, for a four‐state character with state 0,1,2,3 we have

(A24)
$$g_{\max}=\left(t-3\right)c_{0,3}+c_{0,2}+c_{0,1}$$

Proof(s): for *m* see Proof 1, for *g* and *g*_max_, see immediately above and also proof for Type VI binary irreversible characters.

Practical implementation(s): both standard and non‐standard *g* can be found using either the describe n/diag function of PAUP* or the minmax function of TNT, although TNT requires costs to be integers. TNT implements *m*, with the same integer cost restriction. Claddis calculates *m*, *g* and *g*_max_.

### Type VIII—binary “Dollo” (asymmetric) characters

Definition: so‐called “Dollo” characters in cladistic practice often deviate from ideas, attributed to Dollo, of a complex structure never being reacquired once lost, rather encapsulating the related idea that a derived state can only be acquired once (Farris, 1977) or, as considered further below, at most once. A Dollo character has been presented as a cost matrix in which the forward transitions or gains (e.g. state 0 to 1) in the upper cost matrix have an “arbitrarily large” cost “guaranteeing that only one transformation to each derived state will be permitted” (Fig. A1 Type VIII; redrawn from Swofford and Olsen, 1990, their fig. 11). This cost was denoted *M* by Swofford and Olsen (1990) but here we denote it *D*, for Dollo penalty, to avoid possible confusion with the ensemble minimum steps (which is often denoted *M*). Swofford and Olsen (1990) state that the use of a cost matrix of this type is equivalent to the Dollo parsimony algorithm (of Farris, 1977 cited by Swofford and Olsen, 1990). However, we note that a cost matrix definition alone is arguably insufficient to capture some concepts of a Dollo character. Below we use the general algebraic framework for maximum steps, outlined above, to give further mathematical insights into behaviour on the star tree, for a Type VIII Dollo cost matrix, of maximal parsimony steps with different values for this Dollo cost penalty for forward transitions (i.e. gains), denoted *D*.

If the forward transition cost is infinity, a Dollo cost matrix of this type would be the transpose of a standard irreversible character cost matrix and could therefore be considered a type of irreversible character (see Type VI, above). In this case, the mathematical arguments outlined above for a comparable irreversible character would apply to Dollo characters of this definition, with minor alteration of state labelling. For binary Dollo characters we may first designate the state labels such that the transition from state 0 to the “derived” state, 1 has a cost approaching infinity. As in Proof 7 for irreversible characters, on a star tree (or conceptual equivalent), it will then always be cheaper for a state which is of infinite cost to produce (here state 1) to be the ancestral state, so that it need not be derived within the tree. All transitions within the tree must then be backward transitions (i.e. losses) e.g. from state 1 to state 0. This is relatable to the idea of Dollo evolution in some, but not all, uses, in that the derived state (here labelled 1) is not derived within the tree and so a condition that it be derived at most once is not violated. We note that application of a cost matrix containing a weight for forward transitions (e.g. Fig. A1 Type VIII) tends, as that weight approaches infinity, to optimize the root state for the sampled tree to the derived state, or for multistate characters the most derived state.

State graph: as with cost matrices, a state graph representation of a Dollo character is not strictly possible, although an approximation is shown in Fig. A1. This is because there is no means of denoting an arc that is only permitted to be traversed at most once.

NEXUS/TNT coding: standard binary Dollo characters are representable using dollo in NEXUS format. It is not straightforward to code non‐standard Dollo characters in NEXUS format or any Dollo character in TNT format, at least under some definitions. However, interested readers should consult Goloboff (1998) for a practical approach to Dollo‐like characters with TNT.

Empirical frequency: extremely rare, with at least one such character found in 1 of 4,467 (<0.1%) datasets. For example, see character 10 of Paterson et al. (2014).

Calculating *m*: the minimum cost for a standard binary Dollo character, i.e. with transitions from state 1 to state 0 equal to 1 (Swofford and Olsen, 1990, their fig. 11) is *m* = 1 (see multistate Dollo characters, Type IX below). This character type is also covered by general algorithm 1.

Calculating *g*: considering the stated aim of Swofford and Olsen (1990) that “only one transformation to each derived state will be permitted”, we outline below an algebraic method to meet this aim, first, in the binary case, by specifically aiming for a value of *D* such that state 1 (the derived state) will be necessarily ancestral on the star tree if its frequency (*y*) is greater than 1. This will guarantee that, on a star tree, no more than one transition can be made to the derived state.

For a frequency of state 1 greater than 1, let the cost of state 0 being ancestral on the star tree be less than that of state 1 being ancestral. So,

if y>1, then $u_{1}<u_{0}$.

For a binary cost matrix of Type VIII, the transition costs are $c_{0,1}=D$ and $c_{1,0}=1$.

Therefore, $u_{0}=c_{0,1}y=\mathrm{Dy}$ and $u_{1}=c_{1,0}x=x$.

If $u_{1}=u_{0}$, x=Dy and so $\frac{x}{y}=D$.

If y=1, we have x=D. Therefore, if D=x, $u_{0}=u_{1}$.

In implementation, if the Dollo penalty *D* for a binary cost matrix of Type VIII is set to *x*, either state 0 or 1 may be ancestral if the frequency of state 1 is 1 and state 1 will be ancestral otherwise. To match the behaviour of PAUP*, on the star tree, *D* can be set marginally less than *x* (e.g. x×0.999) so that if the frequency of state 1 is 1 then state 0 will be ancestral and state 1 will be ancestral otherwise:

If y=1 and D<x, then $u_{0}<x$ and $u_{1}=x$, therefore $u_{0}<u_{1}$.

In PAUP*, after optimization of ancestral states, the final tree length is calculated using costs equivalent to those from a symmetric matrix transposing the lower Dollo cost matrix, i.e. the Dollo cost penalty is secondarily removed for final cost calculation (Swofford, 2003).

For a binary Dollo Type VIII cost matrix, maximum cost is

(A25)
$$g=\min\left(u_{0},u_{1}\right)=\min\left(c_{0,1}y,c_{1,0}x\right)=\min\left(\mathrm{Dy},x\right)$$

If y=1, and we have set *D* = *x*, as discussed above, then for the original Dollo cost matrix including the Dollo penalty *D*,

(A26)
$$g=\min\left(x,x\right)=x$$

Here, $u_{0}=u_{1}$ (as outlined above).

If y>1, and we have set *D* = *x*, as discussed above, we then also have

(A27)
$$g=\min\left(\mathrm{Dy},x\right)=\min\left(\mathrm{xy},x\right)=x$$

and $u_{1}<u_{0}$ (as outlined above).

If *D* is set marginally less than *x* (as discussed above) we have, where *y* = 1

(A28)
$$g=\min\left(D,x\right)=D$$

and $u_{0}<u_{1}$ (as outlined above).

If final costs are then calculated using a symmetric matrix formed from the lower Dollo cost matrix (or equivalent, as discussed above) where y=1 and $c_{1,0}=1$ then we will have

(A29)
$$g=\min\left(y,x\right)=\min\left(1,x\right)=1$$

If, *D* is set marginally less than *x* and *y* > 1, we have

(A30)
$$g=\min\left(\mathrm{Dy},x\right)=x$$

and $u_{1}<u_{0}$ (as outlined above).

Calculating *g*_max_: for a Type VIII cost matrix, *g*_max_ is equal to the highest possible value of *g* (eq. (A29), where *y* = 1 and eq. A30 where *y* > 1) for a given value of *t* with *n* = 2. By eq. (A29), when *y* = 1, *g* = 1. By condition (ii) *y* > = 1. If *t* = *n* = 2, therefore, *y* = 1. Otherwise, if *y* > 1, by eq. (A30), *g* = *x*. By eq. (1), if *y* = 1, *x* = *t* − 1. Therefore, the highest possible value of *x* where eq. (A30) and conditions (i)–(iii) apply is the highest possible integer, between 1 and *t*, that is less than *t* − 1. The highest integer value less than *t* − 1 is given by *t* − 2. Therefore, we have

If *t* = *n*,

$$g_{\max}=c_{1,0}\left(t-1\right)$$

If *t* > *n*,

(A31)
$$g_{\max}=c_{1,0}\left(t-2\right)$$

Proof(s): for *m*, this reduces to the same situation as a Type IV character and the cost matrix with *D* removed means Proof 1 applies. For proofs for *g* and *g*_max_ see algebraic argument above.

Practical implementation(s): standard *m* and *g* can be found using the describe n/diag function of PAUP*. Non‐standard *m* and *g* are not implemented in PAUP* and Dollo characters are not explicitly supported in TNT but see Goloboff (1998). Claddis calculates *m*, *g* and *g*_max_.

### Type IX—multistate Dollo (asymmetric) characters

Definition: by extension of the argument above for binary Dollo characters, we may apply the mathematical arguments of irreversible multistate characters to multistate Type IX Dollo cost matrices, in which the forward transitions (i.e. state gains) in the upper cost matrix have costs approaching infinity. As the Dollo penalty *D* approaches infinity, this will tend to force the root state of the star tree to the most derived state because this is the only state which can produce all the others without multiplication by cost *D*. We can also determine algebraically the value of *D* that achieves, for a multistate Dollo character, the stated aims of Swofford and Olsen (1990), by extension of the algebraic arguments outlined above for binary Dollo characters, as follows.

For a three‐state Dollo character, we interpret the aim that “only one transformation to each derived state will be permitted” (Swofford and Olsen, 1990) by application of the following constraints to optimization on a star tree (or conceptual equivalent). We aim to choose *D* such that state 0 cannot be optimally ancestral if the frequency *y* of state 1 is greater than 1, and neither state 0 nor state 1 can be optimally ancestral (i.e. state 2 must be optimally ancestral) if the frequency *z* of state 2 is greater than 1:

(A32)
$$\text{If}y>1,\text{then}u_{2}<u_{0}\text{and}u_{1}<u_{0}$$

(A33)
$$\text{If}z>1,\text{then}u_{2}<u_{0}\text{and}u_{2}<u_{1}$$

Where these constraints are met, no more than one transition to a derived state could be optimal on the star tree.

For a standard three‐state Dollo cost matrix of Type IX, $c_{0,1}=D$, $c_{0,2}=2D$, $c_{1,0}=1$, $c_{1,2}=D$, $c_{2,0}=2$ and $c_{2,1}=1$. If the frequencies of states 1 and 2 are 1, y=z=1.

If $u_{1}=u_{0}$, then:

(A34)
$$c_{1,0}x+c_{1,2}z=c_{0,1}y+c_{0,2}z$$

So, substituting in the specified state frequencies and transition costs,

(A35)
x+D=D+2D

Therefore,

(A36)
$$\frac{x}{2}=D$$

If $u_{2}=u_{1}$, and z=1 then:

(A37)
$$c_{2,0}x+c_{2,1}y=c_{1,0}x+c_{1,2}z$$

Then, by substitution and rearrangement,

(A38)
x+y=D

Therefore, if D=x+y, then $u_{2}=u_{1}$. Since, x+y (from eq. (A38)) is necessarily greater than x/2 (from eq. (A36), then if D=x+y it is also true that $u_{1}<u_{0}$. Therefore, if D=x+y, then $u_{2}=u_{1}<u_{0}$.

In practice, setting D=x+y for a three‐state Type IX Dollo cost matrix permits where z=1 that either state 1 or state 2 may be ancestral on the star tree:

(A39)
$$u_{0}=c_{0,1}y+c_{0,2}z=\mathrm{Dy}+2D=2x+\mathrm{xy}+2y+y^{2}$$

(A40)
$$u_{1}=c_{1,0}x+c_{1,2}z=x+D=2x+y$$

(A41)
$$u_{2}=c_{2,0}x+c_{2,1}y=2x+y$$

So, here $u_{2}=u_{1}<u_{0}$

Setting D marginally lower than x+y (e.g. $\left(x+y\right)\times 0.999$) permits only that state 1 may be ancestral when z=1: $u_{2}<u_{1}<u_{0}$, meeting the specified constraints (from eq. (A32) and eq. (A33)). Proof 8 To propose and prove a generally applicable formula (equivalently, eqs (A47) and (A48)) for the Dollo cost penalty *D* for a general *n*‐state Dollo cost matrix (of Type IX), we will therefore extend the algebraic arguments above (which considered binary and three‐state characters). We aim to apply the constraint that where the frequency of the most derived state ($f_{n-1}$) exceeds 1, that most derived state will be optimally ancestral on the star tree, relative to the penultimately derived state:

(A42)
$$\text{If}\;f_{n-1}>1,\text{then}\;u_{n-1}<u_{n-2}$$

Here, we prove the proposition that, in general, a value of the Dollo cost penalty *D* which acts as a tipping point to permit no more than one derivation of a derived state on the star tree is given by the sum of frequencies of all states other than the most derived state.

In general, the implied cost if a most derived state is ancestral on the star tree is given by the sum of costs of transition from that most derived state to each other state multiplied by their frequencies:

(A43)
$$u_{n-1}=c_{n-1,0}f_{0}+c_{n-1,1}f_{1}+.,,,.c_{n-1,n-2}f_{n-2}$$

Similarly, the implied cost if the next‐most derived state is ancestral is

(A44)
$$u_{n-2}=c_{n-2,0}f_{0}+c_{n-2,1}f_{1}.,,,.+c_{n-2,n-1}f_{n-1}$$

For a general Type IX Dollo cost matrix the costs of transition between states *i* and *j* (numbered from 0 in increasing order of derivation) in the upper matrix are given by

(A45)
$$c_{i,j}=\left(j-i\right)D\text{where}j>i$$

The costs in the lower matrix are given by

(A46)
$$c_{i,j}=i-j\text{where}i>j$$

We could then substitute these transition costs (on the left‐hand sides of eqs (A45) and (A46)) into the right‐hand sides of the formulae for the implied cost if a given state is ancestral (eqs (A43) and (A44)). It can be seen that the transition costs if the most derived state is ancestral (for which all i>j and $c_{i,j}=i-j$) start at $c_{n-1,0}=n-1$ and decrease by 1 for each successive term in the expression for implied cost (in eq. (A43)). The accompanying state frequencies start at the frequency of the least derived state and increase by 1 in each successive term. The transition costs if the penultimately derived state is ancestral start at $c_{n-2,0}=n-2$ and decrease by 1 for each successive term up to and including the penultimate term. Accompanying state frequencies also start at the frequency of the least derived state and increase by 1, up to the penultimate term, in each successive term. The ultimate term in the expression for implied cost if the penultimate state is ancestral is distinctive in that it will be the only transition cost for that state in the upper part of the cost matrix and therefore including the Dollo cost penalty *D* (by eq. (A45)). Given all this, if we were to perform these substitutions into the equality $u_{n-1}=u_{n-2}$ (eq. (A42)) and then rearrange this equality, it can be seen that subtraction of the right‐hand terms up to the penultimate term from the terms on the left‐hand side will leave on the left‐hand side single instances of each state frequency up to and including the penultimate state and leave on the right‐hand side the Dollo cost penalty *D* multiplied by the frequency of the ultimate state. If the frequency of the ultimate state is 1, we then have

(A47)
$$f_{0}+f_{1}+,\ldots ,+f_{n-2}=D$$

This is equivalent to

(A48)
$$t-f_{n-1}=D$$

where $f_{n-1}$ is the frequency of the most derived state and *t* is the number of taxa. This completes the proof.

*D* should be set marginally lower than $t-f_{n-1}$ to match the behaviour of PAUP* (e.g. $\left(t-f_{n-1}\right)\times 0.999$).

State graph: this character type cannot be easily specified by a single graph. A possible approximation is a digraph with *n* vertices and 2*n* arcs with the special condition that “forward” arcs may only be traversed once each.

NEXUS/TNT coding: standard multistate Dollo characters are representable using dollo in NEXUS format. It is not possible to code non‐standard Dollo characters in NEXUS format or any Dollo character in TNT format. However, interested readers should consult Goloboff (1998) for a practical approach to Dollo‐like characters with TNT.

Empirical frequency: extremely rare, with at least one such character found in 1 of 4,467 (<0.1%) datasets. For example, see character 15 of Paterson et al. (2014).

Calculating *m*: the minimum cost for a standard Dollo character, i.e. with transitions between adjacent states equal to 1 (Swofford and Olsen, 1990, their fig. 11) is given by the number of states minus 1, *m* = *n* − 1, i.e. the same as for a linear ordered symmetric character with *w* = 1 (see Type IV, above) using a transposition of the lower Dollo cost matrix. This character type is also covered by general algorithm 1.

Calculating *g*: maximum cost for a Type IX Dollo cost matrix can be calculated by first calculating the optimal root for the star tree by the procedure outlined above in which the Dollo cost penalty *D* is set marginally lower that the number of taxa minus the frequency of the most derived state (eq. (A48)). We will denote this optimally ancestral state with subscript *a*. Maximum steps is then given by the sum of the transition cost from this ancestral state to each other state, multiplied by the frequency of that state observed for the character considered. We note, as above, that in practice, these transition costs are calculated using a symmetrical matrix made by transposition of the lower Dollo matrix (such that the Dollo cost penalty is used only in optimization of the star tree root and not in calculation of the value of *g*).

(A49)
$$g=c_{a,0}f_{0}+c_{a,1}f_{1}+.,,,.c_{a,n-1}f_{n-1}$$

Calculating *g*_max_: from eq. (A21) for irreversible characters, as the Dollo cost penalty *D* for a Type IX Dollo cost matrix approaches infinity, maximum steps approaches

(A50)
$$\lim_{i\to \infty }g_{\max}=tc_{n-1,0}$$

From eq. (A23), where each character state must be represented at least once, for a Type IX Dollo cost matrix, maximum steps tends towards

(A51)
$$g_{\max}=\left(t-n+1\right)c_{n-1,0}+c_{n-1,1},\ldots ,c_{n-1,n-2}$$

which is the case when the frequency of the least derived state is maximized and all other states have a frequency of 1.

However, if the Dollo cost penalty *D* is set marginally lower than $t-f_{n-1}$ (eq. (A48) above) then in the case where both the most derived and penultimately derived states have a frequency of 1, we root optimally to the penultimately derived state (as outlined above). In this case maximum steps can be calculated, first, using the costs of transition in a symmetric cost matrix made by transposition of the lower Dollo matrix from the penultimately derived state to each other state, when the frequency of the least derived state is maximized:

If $u_{n-2}<u_{n-1}$

(A52)
$$g_{\max}=\left(t-n+1\right)c_{n-2,0}+c_{n-2,1},\ldots ,c_{n-2,n-1}$$

Applying to eq. (A52) the pattern of costs in a Type IX Dollo cost matrix (outlined above) and formulae for standard sums of finite arithmetic series then gives:

(A53)
$$g_{\max}=\left(n-2\right)\left(t-n+1\right)+\frac{1}{2}\left(n-2\right)\left(n-3\right)+1$$

where $u_{n-2}<u_{n-1}$.

If, however, the frequency of the most derived state is greater than 1, this state must be optimally ancestral on the star tree (as outlined above). In that case

(A54)
$$g_{\max}=\left(n-1\right)\left(t-n\right)+\frac{1}{2}\left(n-1\right)\left(n-2\right)$$

where $u_{n-2}>u_{n-1}$.

Comparing eqs (A53) and (A54) shows that if they are equal, then *t* = *n* + 1. Where *t* > = *n* + 1 (expected in the majority of cases) the maximum steps calculated by eq. (A54) exceeds that from eq. (A53). Therefore, where *t* > *n* + 1 for a Type IX Dollo character with *D* set as outlined above (and for which all *n* states must appear at least once among the *t* taxa) it can be calculated using eq. (A54) (and by eq. (A53) where *t* < *n* + 1).

Proof(s): for *m* see Type IV. For proofs for *g* and *g*_max_ see multistate irreversible characters, and extended Dollo character, proof, above.

Practical implementation(s): standard *m* and *g* can be found using the describe n/diag function of PAUP*. Non‐standard *m* and *g* are not directly implemented in PAUP* and Dollo characters are not explicitly supported in TNT but see Goloboff (1998). Claddis calculates *m*, *g* and *g*_max_.

### Type X—multistate custom cost matrix (symmetric) characters

Definition: a character with three or more states where all costs are symmetric, i.e. *c*_*i,j*_ = *c*_*j,i*_, but direct transitions are variable in cost. (If direct transitions are all equal this would by default be a Type III, IV or V character.)

State graph: a connected graph with three or more vertices and variable edge weights. (If all weights were equal this would by default be a Type III, IV or V character.) Cycles are permitted.

NEXUS/TNT coding: the user can define either a character state tree (usertype cstree in NEXUS format, cstree in TNT format) or cost matrix (usertype stepmatrix in NEXUS format, smatrix in TNT format).

Empirical frequency: very rare, with at least one such character found in nine of 4,467 (0.2%) datasets. For example, see character eight of Sumrall and Brett (2002).

Calculating *m*: this character type is covered by general algorithm 1.

Calculating *g*: covered by general algorithm 2.

Calculating *g*_max_: covered by general algorithm 3.

Proof(s): see exhaustive algorithms 1–3 above.

Practical implementation(s): both standard and non‐standard *m* and *g* can be found using either the describe n/diag function of PAUP* or the minmax function of TNT, although TNT requires costs to be integers. Claddis calculates *m*, *g* and *g*_max_.

### Type XI—binary custom cost matrix (asymmetric) characters

Definition: a character with exactly two states where transition costs are asymmetric, i.e. *c*_0,1_ ≠ *c*_1,0_ and neither cost is infinite. (Both costs cannot be infinite under Rules 4 and 6, and if one cost is infinite then the character is by default a Type VI character.)

State graph: a complete simple two‐vertex digraph with arcs of unequal weight. (Equal arc weights define a Type II character and if one arc were missing it would be a Type VI character.)

NEXUS/TNT coding: the user must define a cost matrix (usertype stepmatrix in NEXUS format, smatrix in TNT format).

Empirical frequency: extremely rare, with at least one such character found in 1 of 4,467 (<0.1%) datasets. For example, see character 3 of Gheerbrant et al. (2014).

Calculating *m*: this character type is covered by general algorithm 1.

Calculating *g*: covered by general algorithm 2.

Calculating *g*_max_: covered by eq. (26) and general algorithm 3.

Proof(s): see exhaustive algorithms 1–3 and Proofs 4 and 5, above.

Practical implementation(s): both standard and non‐standard *g* can be found using either the describe n/diag function of PAUP* or the minmax function of TNT, although TNT requires costs to be integers. Only TNT implements *m*, with the same integer cost restriction. Claddis calculates *m*, *g* and *g*_max_.

### Type XII—multistate custom cost matrix (asymmetric) characters

Definition: a character with three or more states where at least one transition is asymmetric, i.e. *c*_*i,j*_ ≠ *c*_*j,i*_, the costs fulfil Rules 1–7 but do not meet the definition of a Type VII character. If the smallest cost is 1, i.e. min(*c*_*i,j*_) = 1, then this is the standard multistate custom asymmetric character.

State graph: a connected simple digraph composed of *n* vertices and at least *n* − 1 arcs where *n* is ≥3 and the digraph is distinct from a Type VII character digraph. Cycles are permitted.

NEXUS/TNT coding: the user must define a cost matrix with usertype stepmatrix in NEXUS (PAUP*) format or smatrix in TNT format.

Empirical frequency: extremely rare, with at least one such character found in 1 of 4,467 (<0.1%) datasets. For example, see character 7 of Gheerbrant et al. (2014).

Calculating *m*: this character type is covered by general algorithm 1.

Calculating *g*: covered by general algorithm 2.

Calculating *g*_max_: covered by general algorithm 3.

Proof(s): see exhaustive algorithms 1–3 above.

Practical implementation(s): both standard and non‐standard *g* can be found using either the describe n/diag function of PAUP* or the minmax function of TNT, although TNT requires costs to be integers. Only TNT implements *m*, with the same integer cost restriction. Claddis calculates *m*, *g* and *g*_max_.
